# Engineering Cyborg Bacteria Through Intracellular Hydrogelation

**DOI:** 10.1002/advs.202204175

**Published:** 2023-01-11

**Authors:** Luis E. Contreras‐Llano, Yu‐Han Liu, Tanner Henson, Conary C. Meyer, Ofelya Baghdasaryan, Shahid Khan, Chi‐Long Lin, Aijun Wang, Che‐Ming J. Hu, Cheemeng Tan

**Affiliations:** ^1^ Department of Biomedical Engineering University of California Davis CA 95616 USA; ^2^ Institute of Biomedical Sciences Academia Sinica Taipei 11529 Taiwan; ^3^ Department of Surgery University of California Davis School of Medicine Sacramento CA 95817 USA

**Keywords:** cellular chassis, hybrid material, hydrogel, nonreplicating bacteria, nonculturable cells, synthetic biology

## Abstract

Natural and artificial cells are two common chassis in synthetic biology. Natural cells can perform complex tasks through synthetic genetic constructs, but their autonomous replication often causes safety concerns for biomedical applications. In contrast, artificial cells based on nonreplicating materials, albeit possessing reduced biochemical complexity, provide more defined and controllable functions. Here, for the first time, the authors create hybrid material‐cell entities termed Cyborg Cells. To create Cyborg Cells, a synthetic polymer network is assembled inside each bacterium, rendering them incapable of dividing. Cyborg Cells preserve essential functions, including cellular metabolism, motility, protein synthesis, and compatibility with genetic circuits. Cyborg Cells also acquire new abilities to resist stressors that otherwise kill natural cells. Finally, the authors demonstrate the therapeutic potential by showing invasion into cancer cells. This work establishes a new paradigm in cellular bioengineering by exploiting a combination of intracellular man‐made polymers and their interaction with the protein networks of living cells.

## Graphical abstract

Synthetic biology has made major strides toward creating programmable bio‐micromachines. Despite these efforts, current living synthetic cells are created primarily using genetic approaches, and they autonomously divide. Here, the authors show how assembling a man‐made polymer matrix inside bacteria creates semi‐living entities termed Cyborg Cells. The Cyborg Cells are programmable, do not divide, preserve essential cellular activities, and gain nonnative abilities.

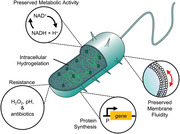

## 1 Introduction

Synthetic biology has made major strides towards the holy grail of fully programmable bio‐micromachines capable of sensing and responding to defined stimuli regardless of their environmental context. A common type of bio‐micromachines is created by genetically modifying living cells.^[^ ^1^ ^]^ Living cells possess the unique advantage of being highly adaptable and versatile.^[^ ^2^ ^]^ To date, living cells have been successfully repurposed for a wide variety of applications, including living therapeutics,^[^ ^3^ ^]^ bioremediation,^[^ ^4^ ^]^ and drug and gene delivery.^[^ ^5^, ^6^ ^]^ However, the resulting synthetic living cells are challenging to control due to their continuous adaption and evolving cellular context. Application of these autonomously replicating organisms often requires tailored biocontainment strategies,^[^ ^7^, ^8^, ^9^ ^]^ which can raise logistical hurdles and safety concerns.

In contrast, nonliving synthetic cells, notably artificial cells,^[^ ^10^, ^11^ ^]^ can be created using synthetic materials, such as polymers or phospholipids. Meticulous engineering of materials enables defined partitioning of bioactive agents, and the resulting biomimetic systems possess advantages including predictable functions, tolerance to certain environmental stressors, and ease of engineering.^[^ ^12^, ^13^ ^]^ Nonliving cell‐mimetic systems have been employed to deliver anticancer drugs,^[^ ^14^ ^]^ promote antitumor immune responses,^[^ ^15^ ^]^ communicate with other cells,^[^ ^16^, ^17^ ^]^ mimic immune cells,^[^ ^18^, ^19^ ^]^ and perform photosynthesis.^[^ ^20^ ^]^ Compared to living cells, however, current nonliving systems have limited biochemical complexities and biological functions owing to the constraints of bottom‐up engineering. Continuing efforts are devoted to advancing synthetic cells for enhanced environmental responsiveness and cell‐like capabilities.

Here, we demonstrate the convergence of both living and artificial systems, creating semi‐living cells with the engineering simplicity of synthetic materials and the complex functionalities of natural cells. We show how hydrogel crosslinking inside bacterial cytoplasm under specific conditions can create nonreplicating yet metabolically active entities, which are herein termed Cyborg Cells. Cyborg Cells preserve genetic material integrity, fluid and functional cell membrane interfaces, and active metabolic pathways. The nondividing property of Cyborg Cells renders them incapable of contaminating the ecosystems like living synthetic cells. Furthermore, Cyborg Cells gain new abilities in resisting stressors that would otherwise kill their unmodified counterparts. Cyborg Cells can be created using different bacterial strains and modified with existing synthetic biology parts readily. Last, we demonstrate the ability of Cyborg Cells to invade cancer cells in vitro. This study showcases how a hybrid approach in the intersection between materials and synthetic biology can result in a cellular platform with nonnatural, engineered, and modular functions, adding to the growing efforts in live cell engineering with synthetic materials.^[^ ^21^, ^22^, ^23^, ^24^, ^25^, ^26^, ^27^, ^28^, ^29^, ^30^, ^31^ ^]^

## 2 Results

### 2.1 Engineering Cyborg Cells through Intracellular Hydrogelation

**Figure 1:**
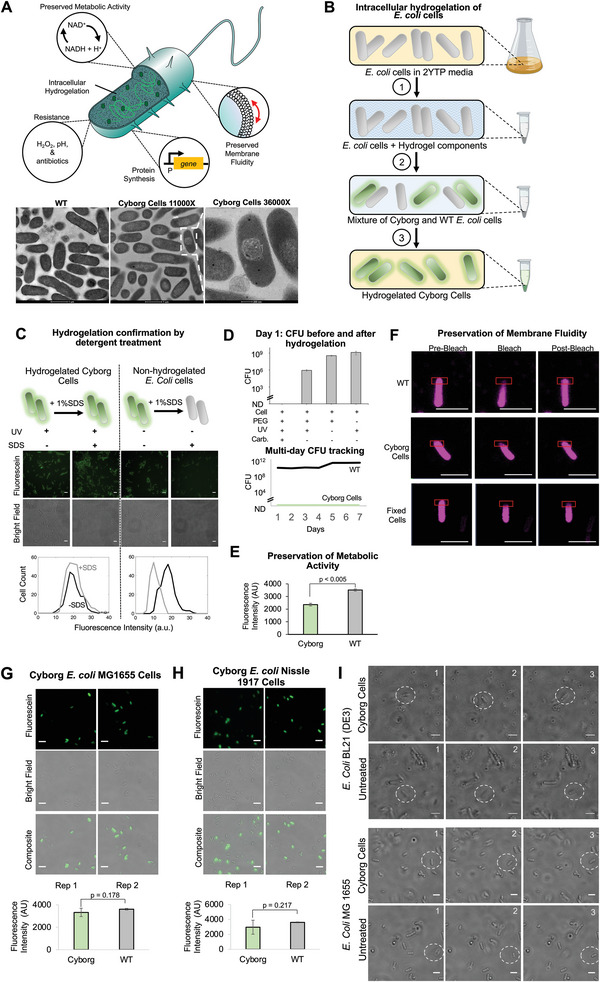
Engineering Cyborg Cells through intracellular hydrogelation. A) Top panel: Graphic representation of a Cyborg Cell highlighting its characteristics. Cyborg Cells do not divide, preserve metabolic and protein‐synthesis activities, maintain membrane fluidity, and gain new resistance to environmental stressors. Bottom panel: TEM images of Cyborg and Wild Type (WT) *E. coli* BL21 (DE3) Cells (Scale bar = 1 µm, zoomed image = 200 nm) (See Methods Section M17). B) Schematic illustrating the procedure to hydrogelate *E. coli* cells. 1) Mix the hydrogel buffer with an exponentially growing culture of the desired *E. coli* strain. 2) Make the hydrogel buffer permeate the bacterial membrane through a freeze and thaw cycle (−80 to 37 °C). 3) Eliminate replicating cells using a high concentration of Carbenicillin. C) Membrane solubilization using 1% SDS to evaluate successful bacterial hydrogelation of *E. coli* BL21(DE3). Top panels: Representative microscopy images of the bacteria infused with hydrogel components treated (+) and untreated (‐) with 1% SDS. Bottom panels: Histogram of single‐cell fluorescence intensity. Non‐hydrogelated bacteria show a decrease in fluorescence intensity caused by the escape of Fluorescein DA after SDS treatment. In contrast, Cyborg Cells maintain green fluorescence intensity after 1% SDS treatment. (Scale bar = 5 µm, *n* = 3 independent experiments). See Methods Section M3, and Figure S1, Supporting Information, for replicates. D) CFU counting assays confirm that Cyborg Cells cannot replicate. Top panel: CFU counts of hydrogelated and non‐hydrogelated bacteria under different conditions on Day 1. Bottom panel: CFU counts of Cyborg Cells and Wild Type bacteria (WT, *E. coli* BL21 (DE3) across 7 days. (Error bar = SD, *n* = 3 independent experiments). E) Cyborg Bacteria Cells preserve metabolic activity. Cyborg Cells created using the strain *E. coli* BL21 (DE3) show comparable levels of metabolic activity according to an assay measuring reduction capacity inside the cell. See Methods M4. Data are presented as mean values. (Error bar = SD, *n*  =  3 independent experiments). Standard two‐tail *t*‐test. F) Fluorescence recovery after photobleaching (FRAP) assay shows the preservation of membrane fluidity in Cyborg Cells. (Scale bar = 2 µm, *n* = 3 independent experiments). See Methods Section M7 and Figure S5, Supporting Information. G&H) Cyborg *E. coli* MG1655 (G) and *E. coli* Nissle 1917 (H) Cells. Top panels: Fluorescence microscopy images of hydrogelated bacteria. The hydrogel was labeled with Fluorescein (Methods Section M2, Figure S6) (Scale bar = 5 µm, *n* = 3 independent experiments). Bottom panels: Metabolic activity of Cyborg & Wild Type cells (Figure S7, Supporting Information) (*n* = 3 independent experiments). I) Cyborg *E. coli* BL21 (DE3) and MG1655 Cells exhibit motility similar to untreated cells. Sequential timelapse images of Cyborg and untreated cells showing similar motility patterns. We followed individual cells across 100 Frames (≈5 s) (See Videos [Link], [Link], [Link], [Link], Supporting Information) (Scale bar = 5 µm, *n* = 3 independent experiments).

We envisioned the creation of a bio‐micromachine chassis with similar capabilities as natural bacterial cells, but with enhanced characteristics provided by their modification with a synthetic material. The chassis would preserve key characteristics of living cells, including cellular metabolism, protein synthesis, membrane fluidity, and functionality of membrane proteins. In addition, the chassis would ideally gain new nonnative functions and would lack the capacity to propagate (**Figure** **1**A).

To create a chassis with the desired traits, we infused bacteria with a chemically stable and nondegradable synthetic hydrogel with low biological reactivity.^[^ ^15^, ^27^, ^32^, ^33^ ^]^ The selected hydrogel chemistry consists of poly(ethylene glycol) diacrylate monomer (PEG‐DA; M*_n_* 700) and 2‐hydroxyl‐4′‐(2‐hydroxyethoxy)‐2‐methylpropiophenone as the photoinitiator. We also incorporated fluorescein *O'O* – diacrylate as a fluorescent dye to check the permeation of the hydrogelation components into the bacteria and the success of intracellular hydrogelation (Figure 1B,C, Methods Sections M2 & M3). The infusion of bacterial cells with the hydrogel components was conducted using a single freeze‐thaw cycle. After the successful infusion of the hydrogel components, the bacterial cells were washed to remove extracellular hydrogel components and cell debris. Crosslinking of the intracellular PEG monomers was triggered using ultraviolet light (UV‐A), which was purposefully chosen to minimize absorption by DNA and other biological components. A 5% hydrogel density was chosen for the intracellular hydrogelation as the particular gel density gives rise to a highly porous scaffold upon crosslinking (Methods Sections M6, Figure S1A, Supporting Information). Hydrogelation was also observed inside the cytoplasm of hydrogelated bacteria under TEM (transmission electron microscopy), and the difference with wildtype bacteria could be observed with hydrogel‐infused Cyborg Cells exhibiting reduced electron transmission (Methods Section M17, Figure 1A, Bottom Panel). After ultraviolet light irradiation, the resulting bacterial cells were incubated in a rich media (37 °C, 250 rpm) and treated with carbenicillin to kill replicating cells. The carbenicillin treatment eliminated bacterial cells that were not successfully hydrogelated, therefore yielding a population of Cyborg Cells unable to divide (Figure 1B). To confirm hydrogelation, we treated both hydrogelated (+UV) and nonhydrogelated cells (‐UV) infused with hydrogel with 1% SDS, an ionic detergent commonly used for the rapid disruption of biological membranes. Hydrogelated cells retained their green fluorescence when compared to nonhydrogelated cells infused with hydrogel (Figure 1C, Figure S1B, Supporting Information). These results are consistent with standard validation assays in the field showing the successful crosslinking of PEG hydrogels. Thus, we confirmed that hydrogel is successfully formed inside bacteria and that hydrogelated bacteria can be identified using dye‐based fluorescence imaging.

Next, we optimized the generation of Cyborg Cells by modifying different parameters, including UV irradiation duration, membrane permeation protocol, and concentrations of crosslinking and culturing reagents (i.e., PEG, photoactivator, and antibiotics). For each perturbation, we characterized the phenotype of the resulting hydrogelated cells to map the key parameters required to produce the nongrowing‐but‐active Cyborg Cells (Figures S2 and S3, Supporting Information). We note that Cyborg Cells could only be generated within a specific hydrogelation window, and nonoptimal conditions resulted in dead cells or non‐hydrogelated cells. To ensure the robustness of our results, we replicated the hydrogelation of bacteria in two different labs at UC Davis (California, USA) and Academia Sinica (Taipei, Taiwan). Repeated generation of the Cyborg Cells suggests that cytoplasmic hydrogel of a particular density allows for biomolecular movements responsible for certain metabolism actions but deprives bacteria of their capability to grow and divide.

Using our optimized protocol, we examined the replication ability of the hydrogelated bacteria using a Colony Forming Unit (CFU) assay (Methods Section M8) and compared the CFU counts obtained from Cyborg Cells, cells incubated with hydrogel components but not treated with UV, and cells treated only with UV (Figure 1D). Before carbenicillin treatment, the CFU of the Cyborg Cell population was ≈1000‐fold lower than the non‐hydrogelated controls. After carbenicillin treatment, the CFU of Cyborg Cells decreased to a nondetectable level. Because our protocol resulted in some non‐hydrogelated bacteria (Figure S3C, Supporting Information), the carbenicillin treatment served to purify the Cyborg Cells population, and Cyborg Cells although nonreplicable, remained active and intact after the treatment (Figure 1E–I). Further experiments demonstrated that over six days of CFU tracking, Cyborg Cells showed no detectable CFUs. Meanwhile, Wild Type cells grew to high‐density levels with daily dilutions (Figure 1D, bottom panel) (for all subsequent results, “Wild Type” denotes the original non‐hydrogelated bacteria). The results confirmed that we can produce a population of intracellular hydrogelated bacteria unable to divide.

### 2.2 The Cyborg Cells Maintain the Active Features of Natural Cells

Furthermore, we characterized the preservation of essential cellular activities after hydrogelation. We assessed the metabolic activity of the Cyborg Cells using a cell viability reagent (PrestoBlue, ThermoFisher Scientific) that reports the reducing power of living cells (Methods Section M4), which is commonly associated with the state of cellular metabolism.^[^ ^34^ ^]^ Our Cyborg Cells exhibited ≈70% of the maximum metabolic activity on the first day, without showing any measurable growth (Figure 1E and Figure S4, Supporting Information). Further experiments showed that our Cyborg Cells preserve quantifiable metabolic activity for up to three days (Figure S7C, Supporting Information), equivalent to ≈150 division cycles of natural bacteria. Living cells preserved constant metabolic activity for the duration of the experiment as expected for a cell population that divides continuously in a rich media. Our results show that the Cyborg Cells preserve reducing power, suggesting the continual functioning of major metabolic pathways.

Another key cellular characteristic that we aimed to preserve in our Cyborg Cells is membrane fluidity, a parameter associated with the correct function and viability of bacterial cells.^[^ ^35^ ^]^ We performed fluorescence recovery after photobleaching (FRAP) assays using a lipophilic DiD dye to assess the state of the lipid membrane in our Cyborg Cells (Methods Section M7). Using this method, we analyzed the membrane fluidity of three different *E. coli* BL21 (DE3) populations: untreated cells, Cyborg Cells, and fixed cells (Figure 1F). Cyborg Cells and untreated cells showed similar recovery halftimes after photobleaching, while fixed cells showed significantly higher recovery halftimes (Figure 1F, Figure S5, Supporting Information).

In contrast to genetic approaches for cell‐chassis engineering, hydrogel‐mediated Cyborg Cell generation could be readily applied to different *E. coli* strains without considering the genetic context. Hence, we tested the intracellular hydrogelation protocol on two different strains with different genotypes and lineages from *E. coli* BL21 (DE3): *E. coli* MG1655 and the probiotic strain Nissle 1917. Consistent with our earlier results, hydrogel was readily established inside Cyborg MG1655 and Cyborg Nissle 1917 Cells under the light‐mediated radical polymerization protocol (Figure 1G,H, top panels, Figure S6, Supporting Information). Furthermore, both strains showed similar metabolic activity to Wild Type cells (Figure 1G,H, bottom panels, Figure S7, Supporting Information), confirming that our hydrogelation protocol can produce Cyborg *E. coli* Cells regardless of the genetic context of the tested strains. Furthermore, Cyborg *E. coli* MG1655 and Cyborg *E. coli* BL21(DE3) Cells showed similar motility to the Wild Type cells (Figure 1I, Videos S1‐S4, Supporting Information, Methods Section M9). Altogether, these results demonstrate that our approach creates hydrogelated bacteria that cannot divide, while preserving membrane fluidity, motility, and metabolic activity.

### 2.3 Inducible Protein Expression and Proteomic Changes in Cyborg Cells

**Figure 2:**
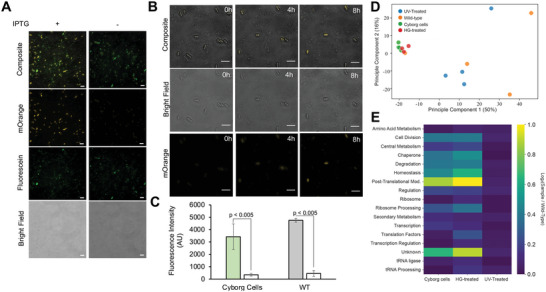
Protein expression and proteome characterization of Cyborg Cells A) Cyborg Bacterial Cells express mOrange in response to IPTG induction. Fluorescence microscopy images of Cyborg Cells derived from the strain *E. coli* BL21 (DE3) pIURKL‐mOrange pLysS after 12 h incubation with and without 1 mM IPTG. (Scale bar = 5 µm, *n* = 3 independent experiments). B) Single‐cell tracking of mOrange expressing Cyborg Cells. Cyborg Cells do not grow but express mOrange after 8 h of IPTG induction. (Scale bar = 5 µm, Methods Section M5). C) Cyborg Cells and Wild Type Bacteria show comparable mOrange expression. Expression levels of mOrange after 12 h incubation with (+, filled bar) and without (‐, unfilled bar) IPTG (Methods Section M11). See Figure S8, Supporting Information, for the continuous tracking of the reaction and for optical density measurements of the samples during this experiment. (Error bar = SD, *n*  =  4 independent experiments). Standard two‐tail t‐test. D) Principal component analysis (PCA) shows the grouping of our different samples based on their protein profile. E) Total log difference between the protein intensities of each sample calculated as the average of each functional group and compared against the abundance of the proteins in our Wild Type control. The colorbar indicates the color code for the value of the total log difference.

We next examined the use of inducible genetic circuits inside Cyborg Cells. First, we created Cyborg Cells using the strain *E. coli* BL21 (DE3) containing a plasmid encoding the fluorescent reporter mOrange under the control of the PT7–lacO hybrid promoter (Methods Section M1). We used this strain to assess if Cyborg Cells retained protein expression capabilities. Specifically, we used fluorescence microscopy (Methods Section M5) and a plate reader (Methods Section M11) to assess if our Cyborg Cells were capable of inducible protein expression (**Figure** **2**A–C). Fluorescence microscopy images showed that Cyborg Cells expressed mOrange in response to IPTG induction (Figure 2A). Moreover, our experiments showed that Cyborg Cells expressed 70% of the total mOrange produced by Wild Type cells despite exhibiting no cellular growth (Figure 2C, Figure S8, Supporting Information). On the contrary, Wild Type cells showed an expected increase in cell density (Figure S8, Supporting Information). Additionally, we used fluorescence microscopy to track the real‐time expression of fluorescent reporter mOrange to further assess for bacterial replication (Figure 2B, Methods Section M5). The results reaffirm the Cyborg Cells’ nonreplicative nature and demonstrate continued protein expression following chemical induction. Additional experiments also demonstrated that our Cyborg Cells express proteins only in the presence of nutrients (Figure S9A, Supporting Information), suggesting that nutrient uptake and metabolism are essential for Cyborg Cells activity. On the other hand, non‐hydrogelated cells incubated under the same conditions replicate in the absence of carbenicillin (Figure S9B, Supporting Information) or die in the presence of carbenicillin (Figure S9C, Supporting Information).

Furthermore, we analyzed the protein composition of our Cyborg Cells through mass spectrometry to identify key proteome changes in response to hydrogelation (Figure 2D,E, Figures S10 and S11, Supporting Information, Methods Section M12). The proteomic content of Cyborg Cells was compared to cell extracts produced from untreated controls (Wild Type Cells), bacterial cells treated with UV (UV‐Treated), and bacterial cells incubated with hydrogel without light‐activated crosslinking (HG‐treated). We used the strain *E. coli* BL21 (DE3) to produce the proteomic extracts of the Cyborg Cells and the controls mentioned above. After standard hydrogelation (Methods Section M2) and processing of the different controls, we performed the digestion, labeling, and tandem mass tag (TMT) mass spectrometry in quadruplicate (Methods Section M12). Mass spectrometry analysis revealed the levels of ≈900 proteins in all samples. After signal normalization (Figure S10A, Supporting Information, Methods Section M12), principal component analysis (PCA) shows clustering and separation of experimental conditions according to the type of sample (Figure 2D). The data show close clustering between Cyborg and HG‐treated cells, whereas Wild Type and UV‐treated cells are clustered separately.

Moreover, we investigated the proteomic changes that are driving the different clustering of each condition. To do this, we plotted the fold change of each protein intensity using Wild Type as our reference and the p‐value from a two‐way t‐test of that comparison (Figure S10B, Supporting Information). According to this analysis, UV‐Treated cells mainly remained unchanged when compared to Wild Type. At the same time, HG‐treated and Cyborg Cells showed statistically significant changes in 29% and 17% of the total number of proteins detected, respectively. To further characterize the proteomic changes, we classified each protein showing a significant change from our Wild Type control into functional groups (Figure S11, Supporting Information). Next, we compared the fold changes between the means of each protein to the Wild Type control and calculated the absolute value of the average of all fold changes in each functional category (Figure 2E). Our analysis indicates that intracellular hydrogelation produces a significant upregulation of proteins involved in homeostasis, protein folding, degradation, central metabolism, and post‐translational modification. In the homeostasis group, notable upregulated proteins include TolC family, RND efflux system, LPS assembly proteins, and Na+/H+ antiporter. In the post‐translational modification group, some notable genes involved in protein assembly were upregulated: BamABCD, SecD, and LolBD. In the cell division group, FtsXNZ involved in the Z‐ring formation was upregulated, but murAC involved in peptidoglycan biosynthesis was downregulated. The analysis shows that our Cyborg Cells have a different proteomic profile than their unmodified counterparts. The proteome profile suggests that the hydrogelation indirectly changes the composition of proteins involved in metabolism, protein synthesis, and protein assembly. Additionally, the proteome changes of the cell division functional group suggest that Cyborg Cells could be partially impeded from dividing due to the downregulated biosynthesis of membrane components. Future work could investigate if the physical impact of intracellular hydrogelation on cellular volume occlusion, DNA replication, and cell segmentation also cause the inability of Cyborg Cells to divide.

### 2.4 Cyborg Cells are Compatible with Synthetic Genetic Circuits

**Figure 3:**
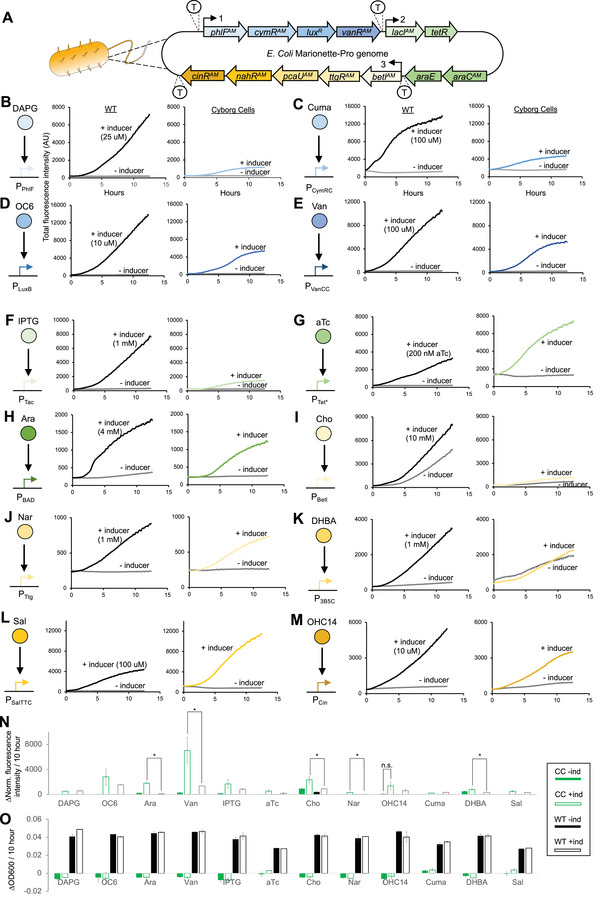
Cyborg Cells can be functionalized using synthetic biology parts. A) Schematic of the Marionette‐Pro strain and its sensor array. B‐M) Response of Wild Type Controls (Left Panel) and Cyborg Cells (Right Panel) to the small molecule activating YFP expression in each strain. Wild Type and Cyborg Cells uninduced (‐) and induced (+) using: B) 25 µM DAPG (2,4‐diacetylphloroglucinol). C) 100 µM Cuma (Cuminic acid). D) 10 µM OC6 (3OC6‐AHL). E) 100 µM Van (Vanillic acid). F) 1 mM IPTG (Isopropyl‐*β*‐d‐thiogalactoside). G) 200 nM aTc (anhydrotetracycline). H) 4 mM Ara (L‐arabinose). I) 10 mM Cho (choline chloride). J) 1 mM Nar (naringenin). K) 1 mM DHBA (3,4‐Dihydroxybenzoic acid). L) 100 µM Sal (sodium salicylate). M) 10 µM OHC14 (3OHC14:1‐AHL). Each schematic on the left of each plot shows the activation (black arrow) of each promoter by the corresponding small molecule inducer (colored circles). Results are not normalized or adjusted based on their optical density. (*n* = 4 independent experiments). N) Expression rate of each Wild Type and Cyborg Cell strain functionalized with different synthetic circuits. Filled green bar = Cyborg Cells without inducer. Open green bar = Cyborg Cells with inducer. Filled black bar = Wild Type cells without inducer. Open black bar = Wild Type Cells with inducer. CC = Cyborg Cells. WT = Wild Type Cells. (Error bars = SD, *n* = 4 independent experiments). Most uninduced and induced pairs show significant differences, except as indicated (n.s.). Only the significantly different induced expression rates between WT and CC are highlighted (*). O) Optical density changes (OD600nm) over 10 h of each circuit. (Error bars SD, *n* = 4 independent experiments). See Methods Section M10.

To further test the capabilities of the Cyborg Cells, we functionalized them using a library of small molecule sensors from the Marionette Sensor Collection.^[^ ^36^ ^]^ We examined if Cyborg Cells could be rapidly functionalized with different synthetic biology parts and if we could produce active and responsive Cyborg Cells using existing synthetic bacterial strains without further genetic changes. Using the plasmids and strains provided with the Marionette Sensor Collection, we created 12 Marionette Strains responsive to the small molecules 2,4‐diacetylphophloroglucinol (DAPG), cuminic acid (Cuma), 3‐oxohexanoyl‐homoserine lactone (OC6), vanillic acid (Van), isopropyl *β*‐d‐1‐thiogalactopyranoside (IPTG), anhydrotetracycline (aTc), l‐arabinose (Ara), choline chloride (Cho), naringenin (Nar), 3,4‐dihydroxybenzoic acid (DHBA), sodium salicylate (Sal), and 3‐hydroxytetradecanoyl‐homoserine lactone (OHC14) (Methods Section M1). We then hydrogelated them (Methods Section M10) to create Cyborg Marionette Cells that respond to each of the 12 small molecule inducers (**Figure** **3**A). Immediately after hydrogelation, Cyborg Marionette Cells and Wild Type control cells were incubated with and without the appropriate inducer (Figure 3B–M) and the expression kinetics and growth were tracked for 12 h.

The 12 Cyborg Marionette Strains expressed the reporter YFP in response to the appropriate inducers and showed similar response compared to Wild Type controls (Figure 3B–M). All Cyborg Marionette Strains showed increased expression of YFP over time in the presence of each inducer. For nearly all inducers (except aTc and Sal, Figure 3G,L), Wild Type cells resulted in higher total fluorescence intensity of the cell population than Cyborg Cells. This result is expected due to the continuous growth of the Wild Type marionette cells contrary to the lack of growth by the Cyborg Marionette Cells (Figure 3O). To approximate single‐cell performance, we calculated the rate of YFP expression by normalizing fluorescence intensity values using OD600 data and then calculated the difference in the normalized fluorescence data at 0 and 10 h (Figure 3N). All Cyborg Marionette strains showed statistically significant differences (*p*‐value < 0.05) between uninduced and induced populations, except OHC14 (*p*‐value = 0.06). Cyborg Marionette Strains responded to Van, Ara, Cho, Nar, and DHBA at a higher rate than the corresponding Wild Type strains (*p*‐value < 0.05). All Cyborg Marionette strains showed negligible change in OD600, consistent with our earlier results about the non‐growing property of Cyborg Cells (Figure 3O). Using the Cyborg Marionette Strain responsive to l‐arabinose, we indirectly assessed the functionality of the arabinose‐proton symporter encoded by the gene *araE*.^[^ ^37^ ^]^ Both Cyborg Marionette and Wild Type cells showed a similar kinetic response to arabinose induction (Figure 3H), suggesting a functional arabinose transporter in our Cyborg Cells, consistent with our earlier FRAP assay showing a fluid and functional cell membrane (Figure 1F). Altogether, these results show that our Cyborg Cells can be functionalized with a diverse set of synthetic biology parts and readily generated from existing synthetic cells.

### 2.5 Cyborg Cells Gain New Nonnative Functionalities

**Figure 4:**
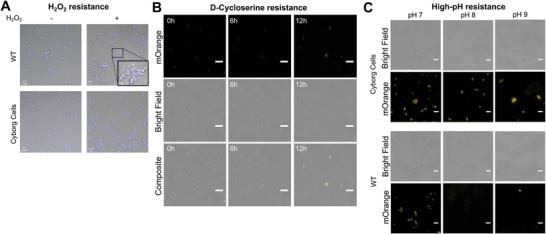
Cyborg Cells gain new non‐native functions A) Cyborg *E. coli* (Migula) Castellani and Chalmers Cells remain stable after hydrogen peroxide (H_2_O_2_) treatment (10% w/w, 3 M). Wild Type Cells are lysed under the treatment with H_2_O_2_. All cells were fixed with 4% paraformaldehyde and then stained with DAPI (10 µg mL^−1^) (Blue color). Representative images (*n* = 3 independent experiments). B) Cyborg *E. coli* BL21(DE3) Cells resist D‐Cycloserine treatment. Wild Type Cells (WT) are lysed. Cyborg Cells remain stable and are capable of mOrange expression when incubated in media containing 200 µg mL^−1^ D‐Cycloserine. (*n* = 3 independent experiments). See Figure S12, Supporting Information. C) Cyborg *E. coli* BL21(DE3) Cells remain stable in media with high pH. Cyborg Cells express mOrange when incubated in media at pH 7–9. Under our experimental conditions, at pH 8, Wild Type Cells form filaments and stop expressing the fluorescent protein reporter. At pH 9, the cells are lysed. (*n* = 3 independent experiments). All cells were induced with IPTG (1 mM) and incubated in a media at the specific pH at the same time. See Figure S13, Supporting Information.

Thus far, we have demonstrated that our Cyborg Cells preserve key functions of living synthetic cells. Next, we tested if the synthetic hydrogel provides new capabilities to the Cyborg Cells. We first examined if the Cyborg Bacteria could resist a hyper oxidative environment containing hydrogen peroxide (H_2_O_2_). H_2_O_2_ is an essential chemical component of host defenses and degradation mechanisms in mammalian cells.^[^ ^38^ ^]^ H_2_O_2_ kills bacterial cells by causing an accumulation of irreversible oxidative damage to the membrane layers, cell wall, proteins, and DNA.^[^ ^39^ ^]^ Using fluorescence microscopy, we tested if Cyborg *E. coli* (Migula) Castellani and Chalmers Cells remained stable in the presence of a lethal concentration of H_2_O_2_ (10% w/w, 3 M) for 3 h at 37 °C and compared the results against Wild Type bacteria (**Figure** **4**A). *E. coli* Migula is commonly used for antimicrobial susceptibility and media testing. After incubation with H_2_O_2_, the morphology and shape of Cyborg Cells remained unchanged, while Wild Type bacteria underwent cell lysis, with fragments and debris observed in microscopy images. These results show that Cyborg Cells remain stable in hyper oxidative environments where Wild Type bacteria cannot survive, indicating that the synthetic hydrogel confers a degree of protection against damaging agents that otherwise will kill natural cells.

In addition, we tested the resistance of Cyborg Cells to cell wall‐targeting antibiotics, D‐Cycloserine (Figure 4B, Figure S12, Supporting Information). D‐Cycloserine is a potent antibiotic particularly effective against gram‐negative bacteria commonly used to treat tuberculosis, and it has a similar mechanism of action to the beta‐lactam class of antibiotics. After hydrogelation, we treated Cyborg *E. coli* BL21(DE3) Cells with D‐Cycloserine (200 µg mL^−1^). We tracked both single‐cell mOrange expression and cell survival for 12 h using fluorescence microscopy (Methods Section M5). Cyborg *E. coli* BL21(DE3) Cells under D‐Cycloserine treatment (Figure 4B) continued to synthesize mOrange protein. In contrast, Wild Type cells lysed during the treatment (Figure S12B, Supporting Information).

Next, we examined Cyborg Cells’ behavior in media with high pH (>pH 7) (Figure 4C). High salinity in both water and soils can cause environments with high pH where only alkaliphiles or alkali‐tolerant bacteria can survive.^[^ ^40^ ^]^ Furthermore, organisms capable of functioning at elevated pH values are deemed essential for biomedical and industrial applications.^[^ ^41^, ^42^ ^]^ Our experiments show that Cyborg *E. coli* BL21(DE3) Cells remained stable and expressed the fluorescent reporter mOrange in response to IPTG induction at pH 7–9 (Figure 4C, Figure S13, Supporting Information). Non‐hydrogelated Wild Type controls behaved similarly to Cyborg Cells at pH7. However, at pH 8, non‐hydrogelated control cells formed filament cells and could not express mOrange. At pH 9, cellular lysis was observed. These results show that hydrogelation confers Cyborg Cells new nonnative capabilities in resisting the stressors, while Wild Type bacteria are killed by the stressors. Cyborg Cells’ robust survival in these stringent conditions presents new opportunities for bioremediation and disease treatment.

### 2.6 Cyborg Cells can Invade Cancer Cells In Vitro

**Figure 5:**
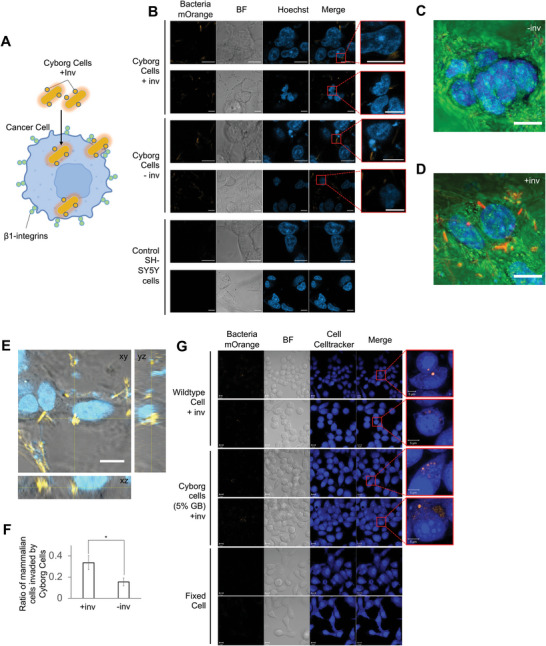
Cyborg Cells are capable of cancer cell invasion A) Schematic of Cyborg Cells expressing mOrange and Invasin (inv+) invading cancer cells. This uptake is facilitated by the binding of invasin and *β*1‐integrins displayed on the membrane of cancer cells. B) Confocal microscopy images of SY‐SY5Y cells coincubated with Cyborg *E. coli* BL21(DE3) Cells expressing (+inv) and not expressing (‐inv) invasin. Controls are SY‐SY5Y cells stained with Hoechst with no Cyborg Cells. All Cyborg Cells express mOrange (orange) and all SY‐SY5Y cells are stained with Hoechst (blue) (Scale bar = 10 µm, *n* = 2 independent experiments). See Methods Section M14. C) Representative image of Cyborg Cells expressing mOrange incubated with SH‐SY5Y cells. The images were obtained through confocal microscopy (Methods Section M14). They were pseudo‐colorized for clarity and to facilitate blind counting of Cyborg Cells invading mammalian cells. (Scale bar = 10 µm, *n* = 2 independent experiments). D) Representative image of Cyborg Cells expressing mOrange and Invasin incubated with SH‐SY5Y cells. The images were obtained through confocal microscopy (Methods Section M14) and pseudo‐colorized for clarity and to help the analysis through blind counting of Cyborg Cells invading mammalian cells. (Scale bar = 10 µm, *n* = 3 independent experiments) E) Cross‐sectional Z‐stack images obtained by confocal microscopy showing Cyborg Cells expressing mOrange and Invasin incubated with SH‐SY5Y cells. Blue: cell nuclei of cells stained with Hoechst dye. Orange: mOrange of Cyborg Cells. Grey: Bright field. See Methods Section M14. (Scale bar = 10 µm, *n* = 2 independent experiments). F) Ratio of SH‐SY5Y cells invaded by Cyborg Cells (inv = Invasin). Error bar = SEM (*n* = 12). See Methods Section M14, and Figure S15, Supporting Information. G) Representative images of Wild Type, Cyborg, and Fixed Cells, incubated with HeLa cells. Methods Section M15, (Scale bars = 10 & 5 µm).

One of the main opportunities of synthetic biology in biomedicine is the delivery of therapeutics by tumor‐invasive bacteria.^[^ ^43^ ^]^ Earlier experiments showed that our Cyborg Cells retain the functionality of their cell membranes and membrane proteins (Figures 1F, 2H). Based on the proven properties of Cyborg Cells, we engineered Cyborg Cells that could invade mammalian cells aided by the protein Invasin (**Figure** **5**A). Invasin is a single‐gene protein from *Yersinia pseudotuberculosis* known to mediate adhesion and invasion of mammalian cells when expressed in *E. coli*.^[^ ^44^ ^]^ Invasin, a 986‐amino acid protein anchored to the outer membrane and encoded by the gene *inv*, promotes uptake into host‐cells by binding to *β*1‐integrins and stimulating Rac‐1.^[^ ^45^ ^]^ As a proof of concept of the therapeutic potential of our synthetic biology chassis, we created Cyborg *E. coli* BL21(DE3) Cells expressing Invasin and mOrange (Methods Section M1, Figure S14, Supporting Information) and tested if the Cyborg Cells could invade cancer‐derived cell lines SH‐SY5Y (neuroblastoma) and HeLa (adenocarcinoma) (Figure 5B–G, Methods Section M14&M15).

Our in vitro experiments confirmed successful cancer cell invasion by Invasin‐expressing Cyborg Cells. Cyborg Cells expressing Invasin and mOrange were coincubated with SH‐SY5Y cells for 4 h (37 °C, 5% CO_2_). After incubation, Cyborg Cells were washed twice and stained with Hoechst dye. Immediately after, all wells containing SH‐SY5Y Cells were imaged using confocal microscopy (Methods Section M14). Confocal microscopy was used to evaluate invasion efficacy as conventional assays that rely on CFU^[^ ^46^ ^]^ are not applicable to the nonreplicative Cyborg Cells. In this experiment, we found distinct differences in the invasion capability of Cyborg Cells expressing Invasin and mOrange versus Control Cyborg Cells only expressing mOrange (Figure 5B–D). Cross‐sectional images show that Cyborg Cells expressing Invasin were located inside the cellular cytoplasm (Figure 5E). In addition, we performed a blind counting test of our microscopy images (Figure S15, Supporting Information, Methods Section M14). This assay shows that Cyborg Cells expressing Invasin and mOrange were able to invade ≈34% of SH‐SY5Y cells as compared to 15% of cellular invasion by the non‐Invasin‐expressing Cyborg Cells (Figure 5F).

In addition, we tested our Cyborg bacteria expressing Invasin and mOrange with HeLa cells, a patient‐derived cell line that has been used to screen for invasive *E. coli* strains. Similar to our previous experiments, Invasin‐expressing Cyborg Cells invaded HeLa cells after a four‐hour incubation (Figure 5G, Methods Section M15). Experiments performed using fixed bacterial cells showed no invasion. Altogether, these results highlight the capacity of Cyborg Cells to act as cancer‐invading systems. However, we note that the invasion efficiency of our Cyborg Cells could be improved and enhanced in future work by fine‐tuning the quantity of Invasin being expressed and with the addition of cancer‐targeting surface proteins.

## 3 Conclusion

Our work demonstrates how the combination of synthetic materials and natural cells can create semi‐living entities with hybrid characteristics and new capabilities. First, we show the infusion of hydrogel components into bacterial cells. Furthermore, contrary to common expectations, careful control of the intracellular assembly of the synthetic polymeric matrix transforms the host bacteria rather than kills them. In addition, we demonstrate that these new synthetic entities maintain key cellular functions such as protein expression, metabolism, and membrane fluidity while becoming unable to divide. The successful test of Cyborg Marionette Strains showcases the potential of our approach to creating Cyborg biosensors. Our hydrogelated bacteria show a different phenotype from their Wild Type counterparts, and the structural support afforded by the synthetic hydrogels likely bestows the Cyborg Cells functional resistance to environmental stressors, including high pH, hydrogen peroxide, and cell wall targeting antibiotics. We hypothesize that this resistance could be driven by the molecular crowding caused by the intracellular hydrogelation of the cytoplasm, thus leading to a state of protective stasis that has been demonstrated naturally in yeast,^[^ ^47^ ^]^ bacterial spores,^[^ ^48^ ^]^ plant seeds,^[^ ^49^ ^]^ and mammalian cells engineered with synthetic polymers.^[^ ^50^ ^]^

Our research establishes a new paradigm in cellular bioengineering by demonstrating an intracellular materials‐based approach to drive living cells to a state of *quasi vita* where they retain and gain certain functions within a limited lifespan. Our work opens the door to new questions about the structure of the hydrogel matrix inside bacteria and its interactions with cytoplasmic proteins and the cellular division machinery. These studies may require additional effort to investigate how the hydrogel affects the cell‐cycle control of bacteria, particularly on the timing control and coordination of replication events. Despite *E. coli* being the best‐characterized model organism, the processes regulating its cell division are not yet fully elucidated. Recent experiments support the hypothesis that cell division is regulated by both replication and replication‐independent events.^[^ ^51^ ^]^ Therefore, we speculate that the hydrogel matrix may be stopping cell division by either suppressing DNA replication, restricting the increase in cell size, or both. To provide a definitive answer, subsequent work may combine Cryo‐EM, high‐resolution microscopy, and proteomic analysis at different stages of the lifespan of the Cyborg Cells to reveal localized protein‐hydrogel interactions. In addition, the protocol can be improved to maximize the number, purity, stability, and activity of the Cyborg Cells.

Altogether, our experiments and these new questions form the basis of a new area that studies the interface between intracellular hydrogels and biomolecules. Research in this area could expand Cyborg Cells for in vivo applications, such as antibacterial treatment, biosensors, gut microbiome modulation, and cancer therapy. The new stress‐resistant feature of Cyborg Cells could allow them to work robustly in certain natural environments. We envision that our Cyborg Cells would become a new class of synthetic therapy‐delivering systems positioned between classical synthetic materials and cell‐based systems. The unique set of characteristics of our Cyborg Cells powered by a combination of synthetic biology, materials science, and bioengineering principles may give rise to a new platform for developing novel biotechnological applications.

## Conflict of Interest

L.E.C‐L., C.M.H., and C.T submitted a provisional patent application covering the process described in this study. The rest of the authors declare no competing interests.

## Author Contributions

L.E.C‐L. and C.T. wrote the paper. L.E.C‐L., C.M.H., and C.T. conceived the work and designed the experiments. L.E.C‐L. performed most of the experiments. Y.‐H.L. performed the experiments in Figures 1F, 4A, and 5G. Y.‐H.L. performed independent validation of the protocols used throughout the paper. T.H. assisted with confocal microscopy and cell invasion tests. C.M. analyzed the mass spectrometry data. C‐L.L. performed Cryo‐SEM experiments. S.K and O.B performed the TEM and flow cytometry experiments. A.W. gave technical advice.

## Supporting information

Supporting Information

Supplemental Video 1

Supplemental Video 2

Supplemental Video 3

Supplemental Video 4

## Data Availability

The data that support the findings of this study are available from the corresponding author upon reasonable request.
