# Supplementary material for: Engineering Cyborg Bacteria Through Intracellular Hydrogelation
Source: Adv Sci (Weinh). 2023 Jan 11;10(9):2204175. doi: 10.1002/advs.202204175 (PMC10037956; doi:10.1002/advs.202204175)
Supplement: Supplementary file 1 — Supporting Information [file ADVS-10-2204175-s001.pdf]

ADVANCED SCIENCE
Open Access

Supporting Information

for *Adv. Sci.*, DOI 10.1002/advs.202204175

Engineering Cyborg Bacteria Through Intracellular Hydrogelation

Luis E. Contreras-Llano, Yu-Han Liu, Tanner Henson, Conary C. Meyer, Ofelya Baghdasaryan, Shahid Khan, Chi-Long Lin, Aijun Wang, Che-Ming J. Hu and Cheemeng Tan**

Engineering cyborg bacteria through intracellular hydrogelation

Luis E. Contreras-Llano[1], Yu-Han Liu[2], Tanner Henson[1,3], Conary C. Meyer[1], Ofelya Baghdasaryan[1], Shahid Khan[1], Chi-Long Lin[2], Aijun Wang[1,3], Che-Ming J. Hu[2]*, Cheemeng Tan[1]*

[1]Department of Biomedical Engineering, University of California, Davis, Davis, CA 95616, USA.
[2]Institute of Biomedical Sciences, Academia Sinica, Taipei 11529, Taiwan.
[3]Department of Surgery, University of California, Davis School of Medicine, Sacramento, CA 95817, USA.

*Corresponding authors: chu@ibms.sinica.edu.tw & cmtan@ucdavis.edu

Supplementary Materials

Section A: Methods

Section B: Supplementary Figures

Section C: Supplementary Movies

Section A: Methods

M1: Construction of plasmids and strains

Plasmids. In this study, we used the plasmids pLysS (Novagen), and pSC101[1] as the backbones for all our constructs. The backbone of the plasmid pSC101 was used to construct the plasmids pIURKL-C.mOrange, and pIURKL. The backbone of the plasmid pLysS was used to construct the plasmids pIURCM and pIURCM-Invasin. All these plasmids have compatible replication origins, distinct copy number, carry a NsiI/PacI cloning site downstream of a PT7–lacO hybrid promoter, and have a T7RNAP terminator sequence. pIURCM and pIURCM-Invasin contains the chloramphenicol resistance gene/p15A replication origin and expresses T7 lysozyme, pIURKL and pIURKL-C.mOrange contain kanamycin resistance gene/pSC101 replication origin. The plasmids pIURCM, pIURKL and pIURKL-C.mOrange were constructed by Villareal et al. [2]. And are available through Addgene [https://www.addgene.org/Cheemeng_Tan/]. The plasmid pIURCM-Inv was generated by PCR amplifying the *inv* gene encoding invasin from *Yersinia pseudotuberculosis* from the plasmid pAC-TetInv [3] (Gift from Christopher Voigt) and inserting it into the PCR amplified backbone of the plasmid pIURCM using Gibson Assembly (New England Bio-Labs, Inc).

The Marionette Sensor Collection [4] was a gift from Christopher Voigt (Addgene Kit #1000000137). Strains obtained from Addgene were used as the source for the plasmids pAJM.711, pAJM.712, pAJM.713, pAJM.714, pAJM.715, pAJM.717, pAJM.716, pAJM.718, pAJM.719, pAJM.1459, pAJM.721, and pAJM.944. All the plasmids from the Marionette Sensor Collection were used without further modification.

Strains. E. coli Top-10 cells (Thermo Fisher) were used throughout this study for plasmid propagation and maintenance. We used the strains *E. coli* BL21 (DE3), *E. coli* Nissle 1917, *E. coli* MG1655, *E. coli* (Migula) Castellani and Chalmers (ATCC 25922™) as the main strains for all our experiments. We created a reporter strain by transforming the plasmids pLysS and pIURKL-C.mOrange into *E. coli* BL21 (DE3). Additionally, we created a cell invasion strain by transforming the plasmids pIURCM-Inv and pIURKL-C.mOrange into *E. coli* BL21 (DE3). The resulting strains are capable of mOrange expression (reporter strain) and the expression of invasin and mOrange (cell invasion strain) to allow mammalian cell invasion and fluorescent reporting in response to IPTG induction. Both strains have resistance to chloramphenicol (34 μg mL^{-1}) and kanamycin (30 μg mL^{-1}).

For Cyborg Marionette experiments we used the strain Marionette-Pro (sAJM.1505; Marionette cluster inserted in the direction of leading strand replication, between 3,720,027 and 3,721,644 in *E. coli* BL21) to transform all twelve plasmids (pAJM.711, pAJM.712, pAJM.713, pAJM.714, pAJM.715, pAJM.717, pAJM.716, pAJM.718, pAJM.719, pAJM.1459, pAJM.721, and pAJM.944) to create Marionette strains responsive to 2,4-diacetylphophloroglucinol (DAPG), cuminic acid (Cuma), 3-oxohexanoyl-homoserine lactone (OC6), vanillic acid (Van), isopropyl β - d-1-thiogalactopyranoside (IPTG), anhydrotetracycline (aTc), l-arabinose (Ara), choline chloride (Cho), naringenin (Nar), 3,4-dihydroxybenzoic acid (DHBA), sodium salicylate (Sal), and 3-hydroxytetradecanoyl-homoserine lactone (OHC14), respectively. All resulting Marionette strains have resistance to low chloramphenicol (5 μg mL^{-1}) and kanamycin (30 μg mL^{-1}).

M2: Intracellular hydrogelation of *E. coli* cells

All the *E. coli* strains used in this study were hydrogelated using the same core protocol with modifications to account for specific requirements of individual synthetic modules or proteins being expressed. Each strain was grown overnight in 3 mL of 2YTP media at 37 °C with shaking at 250 rpm and supplemented, if necessary, with chloramphenicol 34 μg mL^{-1} & kanamycin 30 μg mL^{-1} (Only for *E. coli* BL21 (DE3) transformed with the plasmids pIURKL-mOrange and pLysS, & pIURKL-mOrange and pIURCM-Invasin). For Marionette Strains, we used a concentration of chloramphenicol of 5 μg mL^{-1}. The overnight cultures were diluted 5-fold using 2YTP media with the appropriate antibiotics and incubated for ~2.5 h until the culture reaches an OD of 0.8-1.0. After reaching the appropriate OD, cells are harvested (4000g, 10 min, 20 °C), resuspended in 2YTP media without antibiotics at a cell density of 0.2 g mL^{-1}, aliquoted into 1.5 mL microcentrifuge tubes (ThermoFisher Scientific), and incubated with hydrogelation buffer (1 WT% 2-hydroxy-4′-(2-hydroxyethoxy)−2-methylpropiophenone (Irgacure D-2959; Sigma-Aldrich), and 5% poly(ethylene glycol) diacrylate (PEG-DA; Mn = 700 Da; Sigma-Aldrich) for 30 min at 37 °C with constant rotation at 0.125 Hz on a rotary axis such that the tubes were inverted with each rotation. Fluorescein labeling of the hydrogel polymeric matrix was carried out by supplementing the hydrogelation buffer with 0.1 WT% of fluorescein O,O'-diacrylate (Sigma-Aldrich). After incubation with the hydrogel buffer, bacterial cells were flash frozen by submerging the vials in supercooled methanol at -80 °C for 2 min. Cells were then incubated at -80 °C for 10 min, and then thawed at 30 °C in a dry bath. Vials with bacterial cells were immediately spined down after thawing (6,800g, 10 min, 20 °C), and washed twice using fresh 2YTP media without antibiotics. Bacterial cells infused with hydrogel were then crosslinked with UV light using an energy delivery of 1600 mJ/cm^2 (Light source: UVP Crosslinker CL-3000L - Longwave (365 nm), 115V, Analytik Jena GmbH).

Following UV irradiation, cells were spun down (6,800g, 10 min, 20 °C), and washed twice using 1X PBS buffer. After the final wash and centrifugation, the cells were resuspended and incubated (37 °C, constant rotation at 0.125 Hz on a rotary axis) in 2YTP media with the appropriate antibiotics for each strain, plus carbenicillin (400 μg mL^{-1}) to kill actively dividing, non-hydrogelated bacteria. After incubation, the cells were harvested (6,800g, 10 min, 20 °C) and washed with 1X PBS before being resuspended in 2YTP with the appropriate antibiotics for each strain and carbenicillin (100 μg mL^{-1}) for further experiments.

M3: Detergent treatment for hydrogelation assessment

To assess the successful intracellular hydrogelation of cyborg and wild type cells, we treated different cell populations with 1% Sodium Dodecyl Sulfate (SDS) in PBS buffer to strip cells from their membrane. Briefly, all cells under detergent treatment were washed with PBS buffer twice (6,800g, 5 min, 20 °C) and resuspended in 1% SDS in PBS buffer. Cells were incubated at room temperature with gentle horizontal shaking (60 rpm) for 10 min. Cells were then spined down (6,800g, 5 min, 20 °C) and resuspended in PBS buffer without antibiotics before being imaged using fluorescence microscopy.

M4: Metabolic activity assessment

To assess and compare the metabolic activity of different populations of cyborg and wild type cells, we used the cell permeable resazurin-based PrestoBlue™ Cell Viability Reagent (ThermoFisher Scientific).

After hydrogelation, cells were spined down (6,800g, 5 min, 20 °C) and resuspended in 2YT media with the appropriate antibiotics (chloramphenicol 34 μg mL-1, & kanamycin 30 μg mL-1, & carbenicillin 100 μg mL-1 for hydrogelated bacteria). We plated 90uL of each sample into a 96 well plate (Corning) and diluted the cells with media with the appropriate antibiotics (Final OD600 of 0.1), then we added the PrestoBlue reagent according to the manufacturer specifications to a final concentration of 1X. The fluorescence intensity of the PrestoBlue reagent (Fluorescence top reading, Excitation 560 nm, Emission 595 nm, gain 100) was monitored every 10 minutes for 3 hours (30 °C, double orbital shaking with a frequency of 144 rpms and an amplitude of 2.5 mm, 60s ON, 540s OFF) using an m1000Pro Infinite plate reader (Tecan). Each one of these experiments was compared against a “Wild Type” strain consisting of non-hydrogelated bacteria with the same phenotype as the Cyborg Cells.

M5: Fluorescence microscopy

Microscope images were recorded using a Nikon Eclipse Ti inverted fluorescence microscope with perfect focus 3 (Nikon Instruments Inc) equipped with a 100x/1.4 oil objective. Exposure times were Fluorescein, 300 ms; mOrange 300 ms: Bright Field, 56 ms. Microscope filter settings were Fluorescein, excitation, 450-490 nm; emission, 500-550 nm; gain ≥495 nm. mOrange, excitation, 532-557 nm; emission, 570-640 nm; gain ≥ 565. Samples of cyborg cells, and different bacterial controls were imaged using lab-made 1.5% Low Melting Temperature Agar (LMTA), 1X PBS gel pads. These lab-made gel pads (50 x 25 mm, Thickness: 1 mm) were mounted over glass slides (Plain Micro Slides, 75 x 50 mm, Thickness 1 mm, Corning Inc) and divided in 8 individual squares to allow for imaging of 8 separate samples at once.

Overnight fluorescence microscopy experiments were carried out using similar lab-made 1.5% LMTA, 2YT or 1X PBS gel pads with one isolated square in the center of the gel pad and the rest of the hydrogel arranged to protect the center square from shrinking due to evaporation. Overnight tracking was carried out by taking a 3 x 3 image every 20 minutes (30 °C).

Quantification of fluorescence expression was carried out by measuring the pixel intensity of cyborg bacterial cells and controls in the mOrange fluorescence channel using the open-source platform for biological imaging analysis Fiji (http://fiji.sc/cgi-bin/gitweb.cgi/).

M6: Imaging porous structure of hydrogel using cryo scanning electron microscope (Cryo-SEM)

For obtaining the porous structure image of interior gel, gel was freshly prepared, dissected, and mounted on a stub. The gel was frozen with liquid nitrogen, and then transferred to a preparation chamber at -160°C. As the temperature had reached around -130° C, the sample was etched at -85°C for 15 min. After coating with platinum at -130°C, the sample was transferred to the SEM chamber and under vacuum at -180°C with an acceleration voltage of 20 kV using a cryo scanning electron microscope (FEI Quanta 200/Quorum PP2000TR FEI).

M7: FRAP analysis

Cell membrane was stained by adding 10 µL of DiD dye solution (1,1′-Dioctadecyl-3,3,3′,3′-tetramethylindodicarbocyanine; ThermoFisher Scientific) containing 5 µgmL−1 of DiD dye and 0.5% of DMSO to 200 µL of cell suspension. *E. coli* BL21 (DE3) pLysS pIURKL-mOrange cells were grown overnight and were either hydrogelated or treated with 2.5% glutaraldehyde for 10 min prior to FRAP analysis. FRAP analysis was carried out on a Zeiss LSM780 confocal microscope (Carl Zeiss, Oberkochen, Germany) equipped with Plan-Apochromat 100×/1.4 oil

objective. For FRAP analysis, adherent cells and adherent GCs were used rather than suspension cells to minimize artifacts due to random movements. An objective heater was used to maintain samples at 37 °C. Images were collected with a pinhole of 1.52 AU (1.1 μm section) for optimal signal intensity. The sample was first scanned three times with 5% of laser power to measure the fluorescence intensity before photobleaching, followed by 500 iterative laser pulses at full power to photobleach a 27 nm× 6 nm rectangular area at the plasma membrane. Fluorescence recovery was monitored every 2 s for at least 2 min at 60 frames per second until a plateau is reached. Fluorescence intensity vs. time was plotted for analyzing the fluorescence recovery.

M8: CFU assay

After intracellular hydrogelation of *E. coli* cells, we assessed the proliferation capabilities of the resulting cell population by short and long term CFU assays. Briefly, the resulting cells were diluted 10^{-2}-10^{-8} times and 20 μL were plated in triplicate into individual wells of 24 well plates (Corning) with 2YT agar with the appropriate selection markers. Plates were incubated overnight (37 °C) and the resulting colonies in the appropriate dilutions were counted and reported as CFUs accounting for the dilution factor and the volume used to plate. For accuracy and consistency, we only counted colonies in a dilution if there were between 5-50 colonies. A result of less than 5 colonies was considered as below the detection level.

M9: Motility assay

After intracellular hydrogelation of different *E. coli* populations, we visualized the motility of the resulting Cyborg Cells and their Wild Type controls using Bright Field microscopy. Microscope images were recorded using a Nikon Eclipse Ti inverted fluorescence microscope with perfect focus 3 (Nikon Instruments Inc) equipped with a 100x/1.4 oil objective. Following standard hydrogelation, we resuspended our samples in 1X PBS and standardized the OD600 to 0.1. Next, we took 50 μL of each sample and plated them individually on separate wells of an ibidi 15 μ-Slide Angiogenesis (IBIDI GMBH). After allowing for cells to settle down for 20 minutes, we captured Fast Timelapses of the samples in focus with the maximum frame number (100 Frames). We tracked the movement in the field of view of individual moving cells across each one of the 100 frames and representative frames with tracked cells were selected to illustrate the motility of different samples. Image analysis was carried out using the open-source platform for biological imaging analysis Fiji (http://fiji.sc/cgi-bin/gitweb.cgi/). We assembled videos using all frames of each experiment and the resulting files are showing as supplementary materials for this manuscript.

M10: Response of Cyborg Marionette Cells to different Inducers

We used the Marionette Sensor Collection from Addgene (Addgene Kit #1000000137) [4] to create 12 different strains responsive to the 12 small molecules DAPG, Cuma, OC6, Van, IPTG, aTc, Ara, Cho, Nar, DHBA, Sal, and OHC14. The strains were built by transforming the Marionette protein expression strain Marionette-Pro with each one of the plasmids containing reporters to each one of those 12 small molecules (Methods Section M1). These strains have been shown to express the fluorescent reporter YFP in response to the appropriate inducer.

To create Cyborg Marionette Bacteria, each strain was hydrogelated based on the standard hydrogelation protocol described in “Methods Section M2” with a small variation, the hydrogel polymeric matrix is not labeled with fluorescein due to the fluorescence overlap with YFP (Fluorescein; Excitation max 490 nm, Emission max 525 nm. YFP; Excitation max 513 nm, Emission max 527 nm). Immediately after hydrogelation, cells were spun down (6,800g, 5 min, 20 °C) and resuspended in 2YT media with the appropriate antibiotics (chloramphenicol 5 µg mL^{-1}, & kanamycin 30 µg mL^{-1}, & carbenicillin 100 µg mL^{-1} for hydrogelated bacteria) and if required with the appropriate inducers (Table 1) to an OD600 of 0.1. We plated 100µL of each sample into a 96 well plate (Corning) and the YFP fluorescence intensity (Fluorescence top reading, Excitation 510 nm, Emission 525 nm, gain 100) was monitored every 5 minutes for 12 hours (30 °C, double orbital shaking with a frequency of 120 rpms and an amplitude of 3 mm, 60s ON, 240s OFF) using an m1000Pro Infinite plate reader (Tecan). Each one of these experiments was compared against a “Wild Type” Marionette strain consisting of non-hydrogelated bacteria.

M11: mOrange expression tracking

To track the expression of mOrange by Cyborg and Wild Type Cells, we used the strain *E. coli* BL21 (DE3) pLysS pIURKL-mOrange (Methods Section M1). We plated 100 µL of hydrogelated and Wild Type cells into a 96 well plate (Corning) and the mOrange fluorescence intensity (Fluorescence top reading, Excitation 548 nm, Emission 562 nm, gain 100) and the absorbance at 600 nm was monitored every 5 minutes for 12 hours (30 °C, double orbital shaking with a frequency of 216 rpms and an amplitude of 3 mm, 120s ON, 180s OFF) using an m1000Pro Infinite plate reader (Tecan).

M12: Mass spectrometry

The following protocol was used for initial sample processing, and it was intended to produce a cell free lysate from each one of the samples to facilitate the downstream workflow for mass spectrometry: After hydrogelation (or treatment according to each control, see Note below),

Cyborg and bacterial cells were harvested and washed twice with 1 mL of Buffer A (4000 g, 20 min, 4 °C). Buffer A contains 10 mM Tris-acetate pH 7.6, 14 mM magnesium acetate, and 60 mM potassium gluconate. After the final wash and centrifugation, the pelleted cells were weighed and suspended in 1 mL of Buffer A supplemented with 2 mM DTT (Thermo Fisher Scientific) per 1 g of wet cell mass. To lyse cells by sonication, freshly suspended cells were transferred into 1.5 mL microtubes and placed in an ice-water bath to minimize heat damage during sonication. The cells were lysed using a Q125 Sonicator with a 2 mm diameter probe at a frequency of 20 kHz and 50% amplitude. Sonication was continued for about 27 cycles 10 s ON/10 s OFF. For each 0.5 mL sample, the input energy was ~1000 J. Cell lysates were centrifuged at 12,000 g for 20 min at 4 °C. The supernatant was collected and stored at −80 °C until further use for peptide sample preparation.

The following protocol was used for peptide sample preparation: the proteins in the whole-cell extract preparations were quantified using BCA assay (Thermo Scientific). A volume equal to 250 µg of protein was used for S-Trap (PROTIFI) digestion. Digestion followed the S-trap protocol; briefly, the proteins were reduced and alkylated, the buffer concentrations were adjusted to a final concentration of 5% SDS 50mM TEAB, 12% phosphoric acid was added at a 1:10 ratio with a final concentration of 1.2% and S-trap buffer (100mM TEAB in 90% MeOH) is added at a 1:7 ratio (V/V ratio). The protein lysate S-trap buffer mixture was then spun through the S-trap column and washed 3 times with S-Trap buffer. Finally, 50mM TEAB with 6 µg of trypsin (1:25 ratio) is added and the sample is incubated overnight with one addition of 50mM TEAB with trypsin after 2 h. The following day the digested peptides were released from the S-trap solid support by spinning at 3000 g for 1 min with a series of solutions starting with 50mM TEAB which is placed on top of the digestion solution, then 5% formic acid followed by 50% acetonitrile with 0.1% formic acid. The solution is then vacuum centrifuged to almost dryness and resuspended in 2% acetonitrile 0.1% Triflouroacetic acid and subjected to fluorescent peptide quantification (Thermo Scientific).

The following protocol was used for peptide labeling with TMTs and fractionation: A set of TMTpro-16plex labels were used to label the samples. In total 25 µg of each sample was diluted with 50mM TEAB to 25 µL per replicate. Each sample was labeled with the TMTpro-16Plex Mass Tag Labeling Kit (Thermo Scientific). Briefly, 20 µL of each TMTpro label (126- 134N) was added to each digested peptide sample and incubated for an hour. The reaction was quenched with 1 µl of 5% hydroxylamine and incubated for 15 min. All labeled samples were then mixed and lyophilized to almost dryness. The TMTpro labeled sample was reconstituted, desalted, and

separated into eight fractions by high pH fractionation (Thermo Scientific). One-third of each fraction (~800 ng) was loaded on to the LC-MS/MS for analysis.

The following protocol was used for liquid chromatography and mass spectrometry of the samples: liquid chromatography separation was conducted on a Dionex nano Ultimate 3000 (Thermo Scientific) with a Thermo Easy-Spray source. The digested peptides were reconstituted in 2% acetonitrile/0.1% trifluoroacetic acid and 1 µg in 5 µL of each sample was loaded onto a PepMap 100 Å 3U 75 µm× 20mm reverse-phase trap where they were desalted online before being separated on a 100 Å 2U 50 µm× 150mm PepMap EasySpray reverse phase column. Peptides were eluted using a 120-min gradient of 0.1% formic acid (A) and 80% acetonitrile (B) with a flow rate of 200 nL/min. The separation gradient was run with 2–5% B over 1 min, 5–50% B over 89 min, 50–99% B over 2 min, a 4-min hold at 99% B, and finally 99% B to 2% B held at 2% B for 18 min.

The following protocol was used for mass spectra acquisition: mass spectra were collected on a Fusion Lumos mass spectrometer (ThermoFisher Scientific) in a data-dependent MS3 synchronous precursor selection method. MS1 spectra were acquired in the Orbitrap, 120 K resolution, 50 ms max injection time, 5 × 105 max injection time. MS2 spectra were acquired in the linear ion trap with a 0.7 Da isolation window, CID fragmentation energy of 35%, turbo scan speed, 50 ms max injection time, 1 × 104 AGC, and maximum parallelizable time turned on. MS2 ions were isolated in the ion trap and fragmented with an HCD energy of 65%. MS3 spectra were acquired in the orbitrap with a resolution of 50 K and a scan range of 100–500 Da, 105 ms max injection time, and 1 × 105 AGC.

The following process was followed for peptide and protein identification: identification of peptides and proteins was conducted using the PAW pipeline [5]. In brief, the ProteoWizard toolkit is used to convert the MS scans into intensity values and extract the TMT reporter ion peak heights. The Comet database search engine is then used to identify peptides. The *E. coli* BL21 (DE3) proteome UP000002032 and a list of known contaminants and expressed protein sequences were used for protein identification. Results are filtered based on a desired false discovery rate using the target decoy method. Identified proteins with sequence coverage of <5% were excluded from the downstream analysis.

The following process was used to normalize the TMT results to account for differences in sample loading: The protein intensities from each of the tagged samples were summed and the average of all the sums was calculated. A normalization factor was calculated by dividing the

average sum by the sum of the sample. The normalization factor for each sample was then multiplied by all the protein intensities present.

The following process was used for the assignment of gene ontological function: identified proteins were assigned gene ontological functions based on the gene ontology identifiers provided in the *E. coli* BL21 (DE3) proteome UP000002032. The gene ontology identifiers were grouped based on the general functional categories of interest. Proteins that lacked identifiers or only possessed broad identifiers were classified as Unknown. Several proteins with known functions that lacked identifiers were manually assigned an appropriate functional group.

Note: The control cells for this experiment were obtained by following the standard hydrogelation procedure described in Methods Section M2 but omitting steps or incubation with certain reagents to create non-hydrogelated cells subjected to only specific parts of the hydrogelation procedure. The "UV-Treated control" cells were obtained by omitting the incubation with hydrogel buffer and the incubation with a high concentration of Carbenicillin. The "HG-treated control" cells were obtained by omitting the crosslinking with UV light and the incubation with high concentration of Carbenicillin. The "Wild Type control" cells were obtained by omitting the incubation with hydrogel, the exposure to UV light and the incubation with high concentration of carbenicillin.

M13: SDS-PAGE analysis

Analysis of proteins by SDS-Polyacrylamide Gel Electrophoresis (PAGE) was carried out by separating proteins from whole-cell lysates and CFPS reactions using 4-20% Mini-PROTEAN TGX precast gels (Bio-Rad). We used Precision Plus Protein Dual Color Standards (10–250 kDa) as a reference standard for molecular weight verification. Protein gels were endpoint stained using PageBlue Protein Staining Solution (Thermo Fisher Scientific) according to the manufacturer instructions. Gels were imaged using a PXi Imaging system (Syngene) and band analysis and protein quantification were carried out using the open-source platform for biological imaging analysis Fiji (http://fiji.sc/cgi-bin/gitweb.cgi/) and the proprietary software GeneTools (Syngene).

M14: Invasion of SH-SY5Y cells by Cyborg and wildtype *E. coli* cells

We used SH-SY5Y Cells (CRL-2266™; SH-SY5Y cells are a thrice cloned subline of the neuroblastoma SK-N-SH line derived from a metastatic bone tumor) as a model for mammalian cancer cells for invasion experiments using cyborg *E. coli* cells. In this experiment, we used *E. coli* BL21 (DE3) cells transformed with the plasmids pIURCM-Inv and pIURKL-C.mOrange induced overnight with IPTG to create *+mOrange +Inv* cyborg cells. Our negative controls were produced using *E. coli* BL21 (DE3) cells transformed with the plasmids pLysS and pIURKL-

C.mOrange induced overnight with IPTG to create +mOrange -Inv cyborg cells. Both varieties of cyborg cells were produced using to the protocol described in the section "M2: Intracellular hydrogelation of *E. coli* cells".

SH-SY5Y cells were expanded for said experiments by first taking aliquot of cells from liquid nitrogen tanks and thawing in 37 °C bead bath until thawed. Immediately, 1 mL of cells were resuspended in 4 mL of D5GF media and centrifuged at 1400 RPM for 5 minutes. D5GF media comprised of DMEM High Glucose, Fetal Bovine Serum, Epidermal growth factor, fibroblast growth factor, and penicillin-streptomycin. After centrifugation, cell waste was aspirated, and the cell pellet was resuspended in 2 mL of media. A 20 μL aliquot was taken and mixed with 20 μL of trypan blue and counted on a hemocytometer. Live cell averages of the 4 corners of the hemocytometer were taken and used to calculate total live cell count. Cells were then plated on a T25 flask at a density of 5, 000, 000 cells. Cells were grown to confluency with media changes every other day then passaged. The cells were removed from the plate surface by first washing with PBS, then incubating with trypsin for 5 minutes. Trypsin was inactivated using penicillin-streptomycin free D5GF. Cells were centrifuged and counted in the same manner as before. The SH-SY5Y cells were plated on an ibidi μ-Slide 8 Well high Glass Bottom (IBIDI GMBH) at a density of 50,000 cells per well with penicillin-streptomycin free D5GF. Cells were incubated at 37 °C 5% CO_2 for at least 1 day. After 2 days, the cells were washed with pen-free D5GF and incubated with cyber bacteria at a normalized optical density of 0.05 and 0.1 at 37 °C 5% CO_2.

After the 4 h incubation with the cyborg and the Wild Type bacterial cells, SH-SY5Y Cells were washed twice using 1X PBS buffer and stained with Hoechst dye (20 mM, 15 min) for staining of DNA and nuclei of the mammalian cells. Immediately after, all wells containing SH-SY5Y Cells were imaged using confocal microscopy (ZEISS LSM800, 63x objective) and the image analysis was done using ZEISS ZEN Software. For the experiments conducted with the Zeiss Confocal Microscope, mOrange was imaged using an excitation of 546 nm, an emission of 562, a detection wavelength range of 535-700, and a pinhole of 0.83 AU with a laser wavelength of 561 nm at 2.01%. Hoechst was imaged using excitation of 353 nm, emission of 465 nm, a detection wavelength of 400-545/550 and a pinhole of 0,76 AU with a laser wavelength of 405 nm at 0.2%. All experiments were carried out in duplicate with three technical replicates each.

Blind counting of Cyborg Cells invading mammalian cells. To assess the invasion of mammalian cells by cyborg cells, we selected 24 images, (6 representative images per condition), and these were re-named and randomized. The brightfield of the images was artificially colored in green to help with contrast. Two volunteers were selected and were told what each of the stains

represented, such as blue for Hoechst, green for brightfield, orange for bacterial cells expressing mOrange. Three example images of invasion and one of non-invasion were shown and explained to each of the volunteers. Individually, the volunteers went through all images and were asked to count number of mammalian cells, number of mammalian cells with at least one bacterium invaded, and total number of bacteria invaded. These numbers were recorded by the volunteers and were hidden from the other volunteer.

M15: Invasion of HeLa cells by Cyborg *E. coli* cells

In this experiment, we used *E. coli* BL21 (DE3) cells transformed with the plasmids pIURCM-Inv and pIURKL-C.mOrange induced overnight with IPTG to obtain *+mOrange +Inv* wildtype, cyborg and fixed cells. Cyborg cells were produced using to the protocol described in the section "M2: Intracellular hydrogelation of *E. coli* cells".

HeLa cells (CCL-2™; HeLa cells are cervical carcinoma cell line derived from a patient) were grown in a media comprised of DMEM (Life Technologies, cat. 11995065), Fetal Bovine Serum (FBS), and Penicillin-Streptomycin (PS, Life Technologies, cat. 15140-122) and plated on a confocal dish at a density of 2×10^5 cells/well. The cells were grown overnight at 37°C, with 5% CO_2. The culture medium was removed. Cells were stained with 20 µM of Celltracker Blue CMAC dye (Invitrogen, CA) in DMEM only (without FBS and PS) for 0.5 hour. After 0.5 h incubation, the DMEM was removed and fresh DMEM was added (with FBS and PS). Bacteria, controls, and Cyborg bacteria were spun down at 4000g for 10 minutes, and the pellet was resuspended in the Penicillin Streptomycin absent media and mixed with Kanamycin (1:1000 dilution) (VWR cat. 25389-94-0), Chloramphenicol (1:3000 dilution) (AMRESCO cat. 56-75-7), Carbenicillin (1:1000 dilution) (Cyborg bacteria only) (Millipore Sigma cat. C3416-5G), and 1 mM IPTG. The dosing of the bacteria and Cyborg bacteria was performed using 20 µL of the bacteria, hydrogelated bacteria, or fixed bacteria-media mixture and added to plates containing the mammalian cells placed in the 37°C, 5% CO_2 incubator. The bacteria were incubated for 4 hours together with the mammalian cells. After this incubation, the wells were washed twice with DMEM and then washed once with PBS (Fisher Scientific, cat. BP243820). The cells were kept in 4% paraformaldehyde/PBS for imaging. Imaging was performed using confocal microscopy (ZEISS LSM880, 63x objective) and image analysis using the software ZEISS ZEN.

M16: Statistical Analysis of results

Statistical tests were performed using standard two-tailed t-test, assuming unequal variances. Significant results were defined as those with p-values less than 0.05 and were indicated in each

figure by adding an asterisk. The number of replicates contributing to the calculation is listed in the figure legends.

M17: Sample preparation for TEM microscopy

Wild type and Cyborg *E. Coli* BL21 (DE3) cells were hydrogelated, and the pellets were fixed in a fixative solution (2.5% glutaraldehyde, 2% paraformaldehyde in 0.1 M sodium phosphate buffer). After fixation, samples were rinsed twice in 0.1 M sodium phosphate buffer for 30 min and placed in 1% osmium tetroxide in 0.1 M sodium phosphate buffer for 1 hour. Samples were rinsed 2 times for 15 minutes each in 0.1 M sodium phosphate buffer. The samples were then dehydrated in 50% EtOH, 75% EtOH, 95% EtOH (at least 30 min each), and finally in 100% EtOH (2 x 20 min). Samples were placed in propylene oxide 2x15 minutes and were pre-infiltrated in half resin/ half propylene oxide overnight. The next day samples were infiltrated in 100% resin (composed of 450 ml dodecenyl succinic anhydride, 250 ml araldite 6005, 82.5 ml Epon 812, 12.5 ml dibutyl phthalate, and 450 μl benzyldimethylamine) for 5 hours. The samples were embedded with fresh resin and polymerized at 65°C overnight. The embedded cells were sectioned with a Leica EM UC6 ultramicrotome at a thickness of 90 nm and collected on copper mesh grids. The sections were stained with 4% aqueous uranyl acetate for 20 minutes and for 2 minutes in 0.2% Lead Citrate in 0.1 N NaOH. TEM imaging was done on FEI Talos L120C at 80kv using Thermo Scientific Ceta 16MP camera.

M18: Sample preparation for Flow Cytometry analysis

Wild type and Cyborg cells were hydrogelated, and pellets were resuspended in 1X PBS. OD of the bacteria was normalized to 0.1 and samples were analyzed on Beckman CytoFLEX S flow cytometer with 488 nm blue laser excitation coupled with 525/40 nm bandpass filter.

Supplementary References

[1] D. Manen, L. Caro, *Mol. Microbiol.* **1991**, *5*, 233.

[2] F. Villarreal, L. E. Contreras-Llano, M. Chavez, Y. Ding, J. Fan, T. Pan, C. Tan, *Nat. Chem. Biol.* **2018**, *14*, 29.

[3] J. C. Anderson, E. J. Clarke, A. P. Arkin, C. A. Voigt, *J. Mol. Biol.* **2006**, *355*, 619.

[4] A. J. Meyer, T. H. Segall-Shapiro, E. Glassey, J. Zhang, C. A. Voigt, *Nat. Chem. Biol.* **2019**, *15*, 196.

[5] P. A. Wilmarth, M. A. Riviere, L. L. David, *J. Ocul. Biol. Dis. Infor.* **2009**, *2*, 223.

Section B: Supplementary Figures

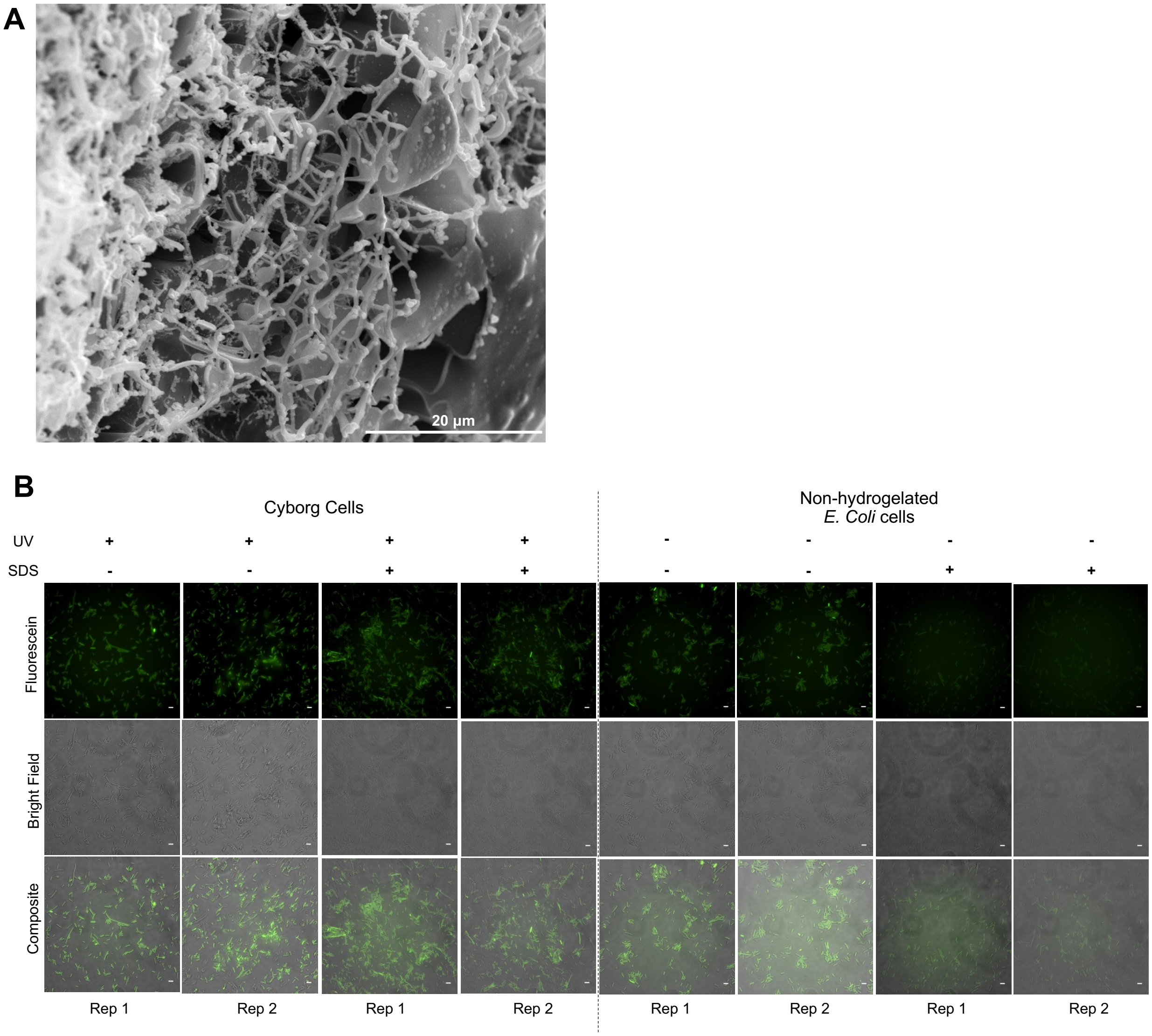

Supplementary Figure 1: Characterization of the PEG-700 diacrylate hydrogel matrix *in vivo* and *in vitro.*

A) CryoSEM image of the loose porous structure of hydrogel scaffold generated by 5% PEG diacrylate without cells. The image of the gel itself reflects the same hydrogel crosslinking density as that for the cyborg cell preparation (Scalebar = 5μm. See Methods Section M6). **B**) Replicate images of the SDS detergent treatment of Cyborg and non-hydrogelated *E. coli* cells. (Scale bar = 5μm. See Methods Section M3).

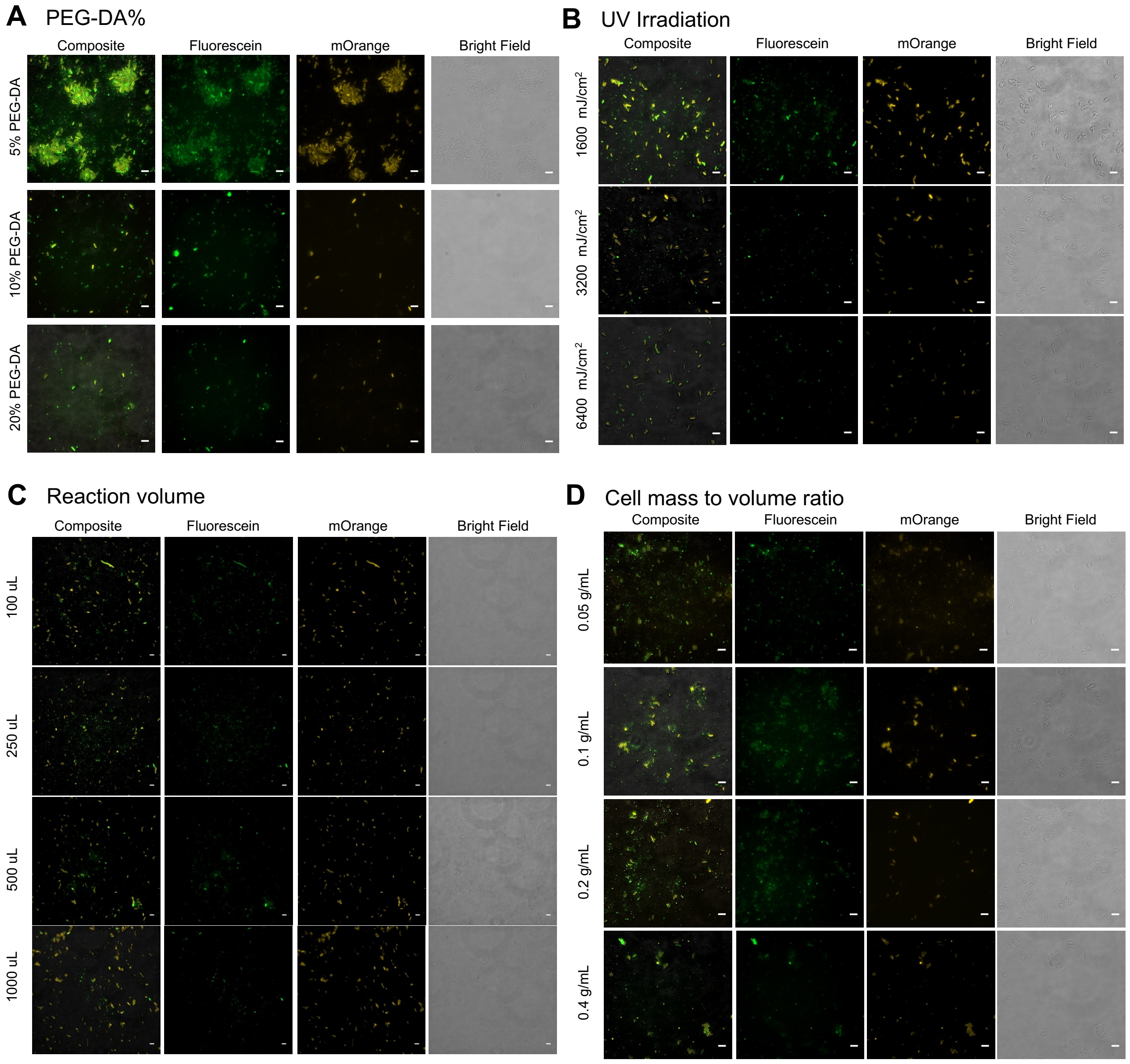

Supplementary Figure 2: Optimization of the intracellular hydrogelation protocol

Optimization of: **A)** PEG-DA concentration **B)** UV irradiation energy **C)** Reaction volume **D)** Cell mass to volume ratio. Representative images of 3 independent experiments. Scale bar = 5μm.

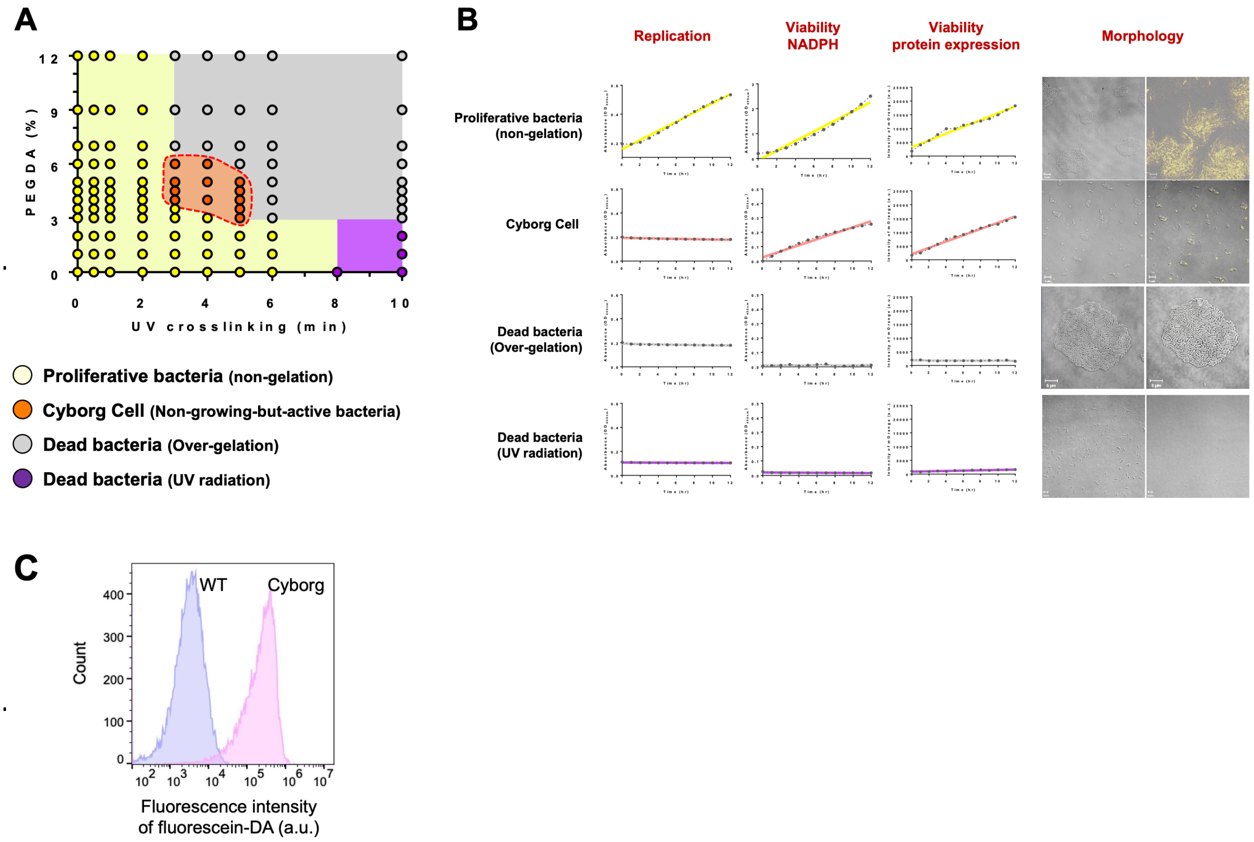

Supplementary Figure 3: Cyborg Cells are generated under optimal hydrogelation conditions

A) Optimal PEG-DA% and UV crosslinking time. Orange region highlights the parameters that generate Cyborg *E. coli* BL21 (DE3) Cells. Hydrogelation conditions were screened in 96-well plates.

B) Metrics used for identifying Cyborg Cells. Cyborg cells should not replicate (first column) but should preserve metabolic activity (2nd column, NADP reduction to NADPH) and protein-synthesis (3rd column) activities. mOrange expressing *E. coli* BL21(DE3) cells under the different conditions listed were characterized to obtain a characteristic phenotype for Cyborg Cells using four different parameters: Cell division, metabolic activity, and protein expression, and characteristic morphology. Scale bar = 5μm.

C) Flow cytometry analysis of Cyborg and Wild type *E. coli* BL21 (DE3) bacteria. The histogram shows the intensity of fluorescein O'O – diacrylate in Wild type (purple) vs. Cyborg (pink) bacteria. (See Methods Section M18).

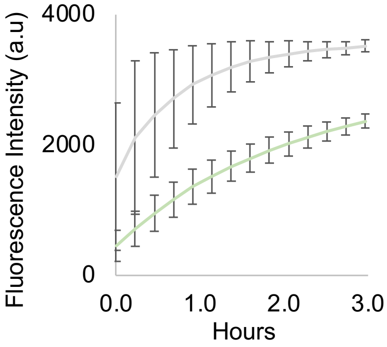

Supplementary Figure 4: Metabolic activity of Cyborg *E. coli* BL21 (DE3) Cells

Metabolic activity of Wild Type and Cyborg *E. coli* BL21 (DE3) Cells. See Methods Section M4. Error bars = SD (n=4 independent experiments).

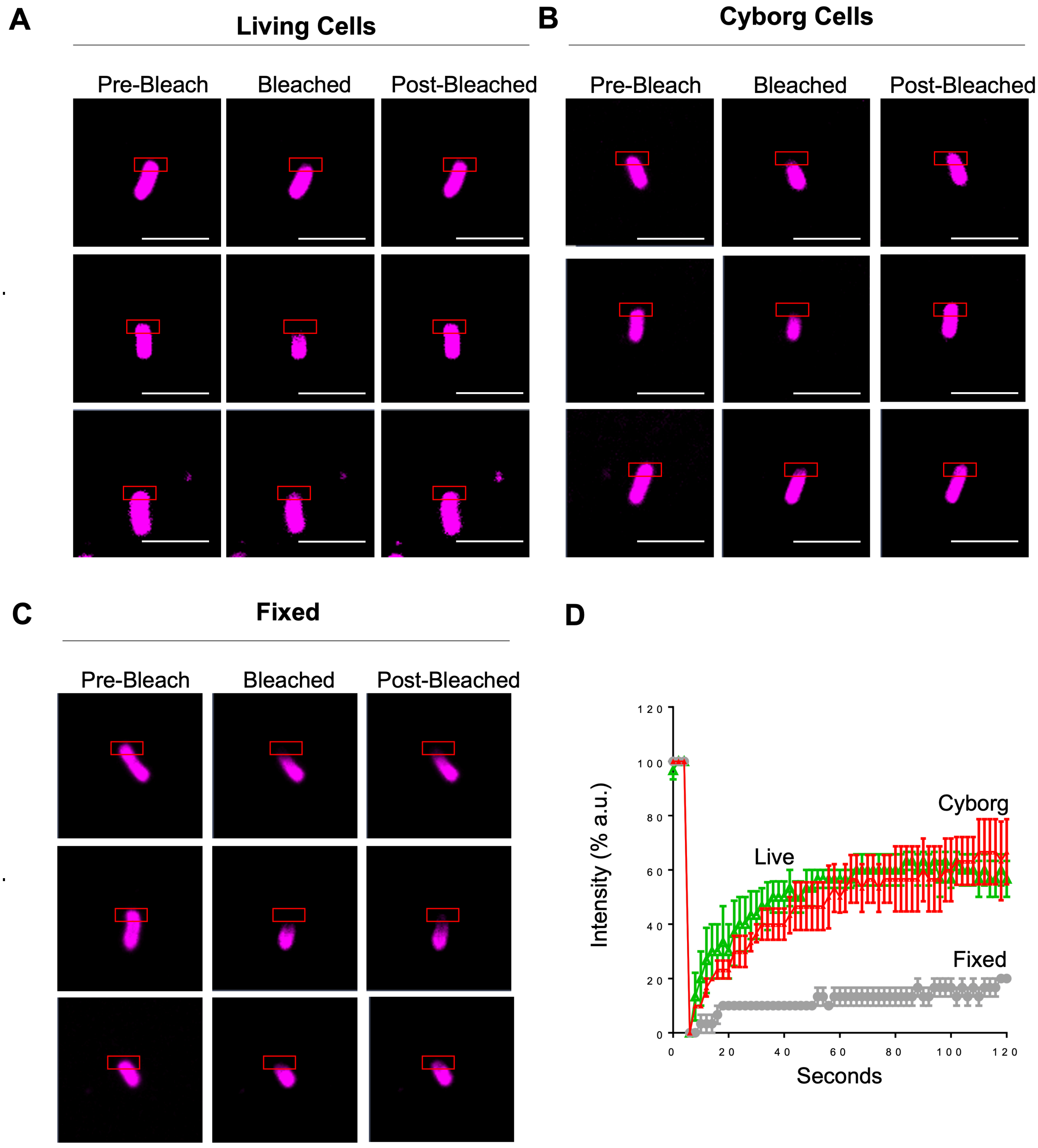

Supplementary Figure 5: Replicates of the FRAP assays on Living, Cyborg and Fixed Cells

A) Images of the FRAP experiments performed in living *E. coli* cells showing the Pre, Post and Bleached stages of the experiment.

B) Images of the FRAP experiments performed in Cyborg *E. coli* cells showing the Pre, Post and Bleached stages of the experiment.

C) Images of the FRAP experiments performed in fixed (Non-hydrogelated) *E. coli* cells showing the Pre, Post and Bleached stages of the experiment. Scalebar = 2μm.

D) FRAP curves showing the percentage of fluorescent intensity (a.u.) compared to the pre-Bleached samples of Live (green line), Cyborg (red line), and Fixed (grey line) cells. Images were taken at timepoints 0s (Pre-Bleach), 8s (Bleached), and 120s (Post-Bleached). See Methods Section M7. Error bars = SEM (n= 3 independent experiments)**.**

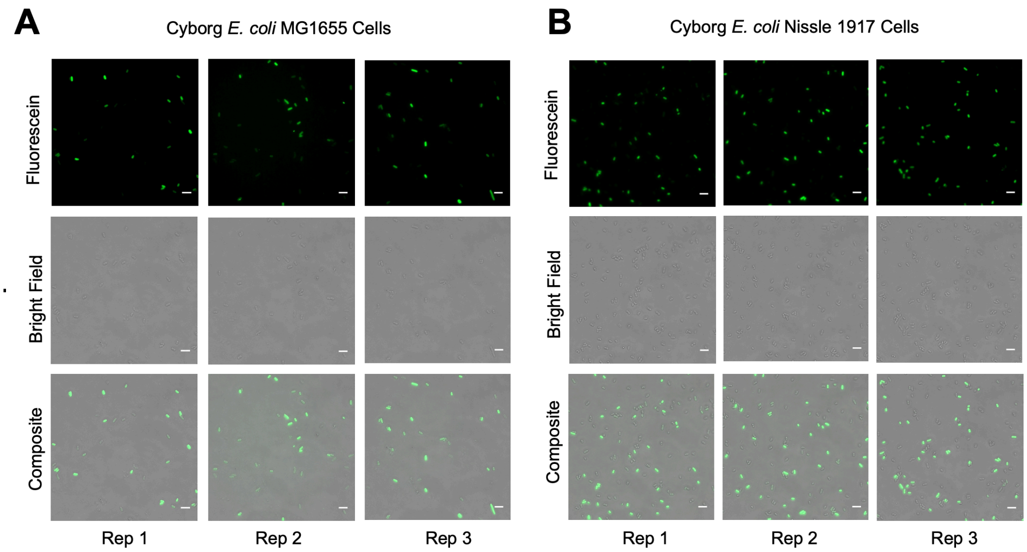

Supplementary Figure 6: Replicates showing the creation of Cyborg *E. coli* MG1655 & Nissle 1917 cells

Fluorescence microscopy images of: **A)** Cyborg *E. coli* MG1655 cells, and **B)** Cyborg *E. coli* Nissle 1917 cells. (n= 3 independent experiments). See Methods Section M2. Scalebar = 5 μm.

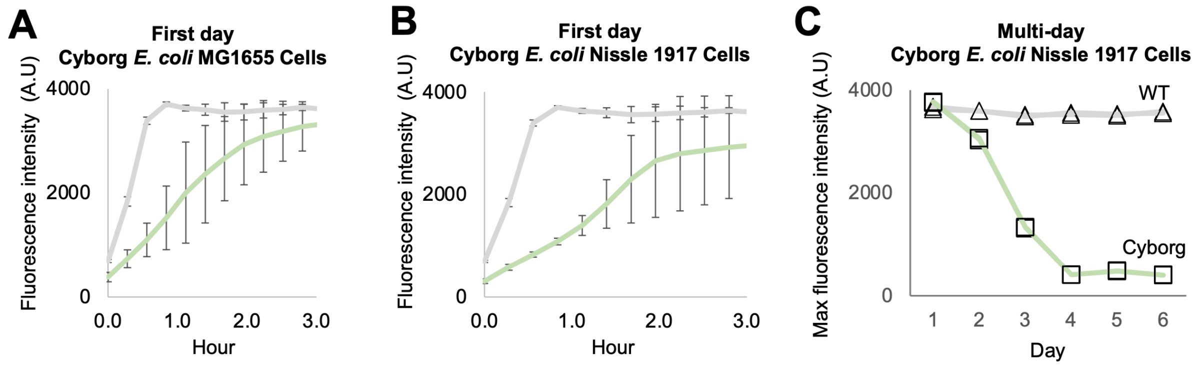

Supplementary Figure 7: Metabolic activity of Cyborg *E. coli* MG1655 & Nissle 1917 cells

A&B) Metabolic activity of **A)** Wild Type and Cyborg *E. coli* MG1655 cells, and **B)** Wild Type and Cyborg *E. coli* Nissle 1917 cells right after hydrogelation. See Methods Section M4. Error bars = SD (n=4 independent experiments). **C)** Multi-day tracking of the metabolic activity of Wild Type and Cyborg *E. coli Nissle* 1917. The maximum fluorescence intensity of each daily assay is shown. Results are not normalized or adjusted based on their optical density.

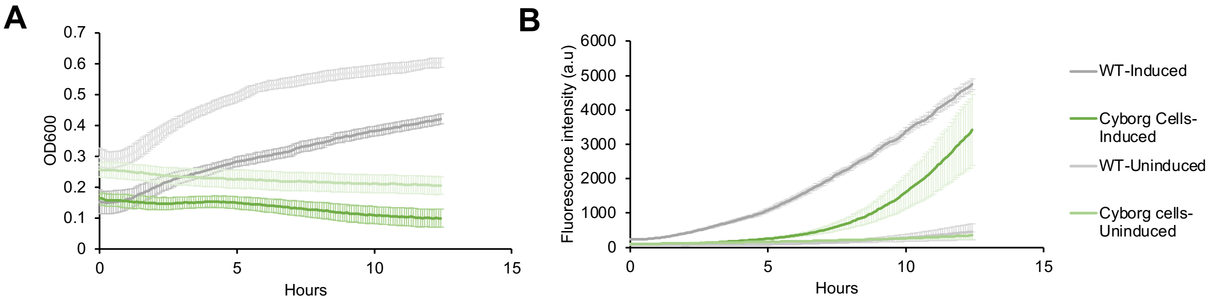

Supplementary Figure 8: Tracking of Cyborg *E. coli* BL21 (DE3) Cells expressing mOrange

A) OD600 tracking of Wild Type and Cyborg *E. coli* BL21 (DE3) Cells induced and uninduced to express mOrange. **B)** Fluorescence tracking of Wild Type and Cyborg *E. coli* BL21 (DE3) Cells induced and uninduced to express mOrange. See Methods Section M11. Error bars = SD (n=4 independent experiments).

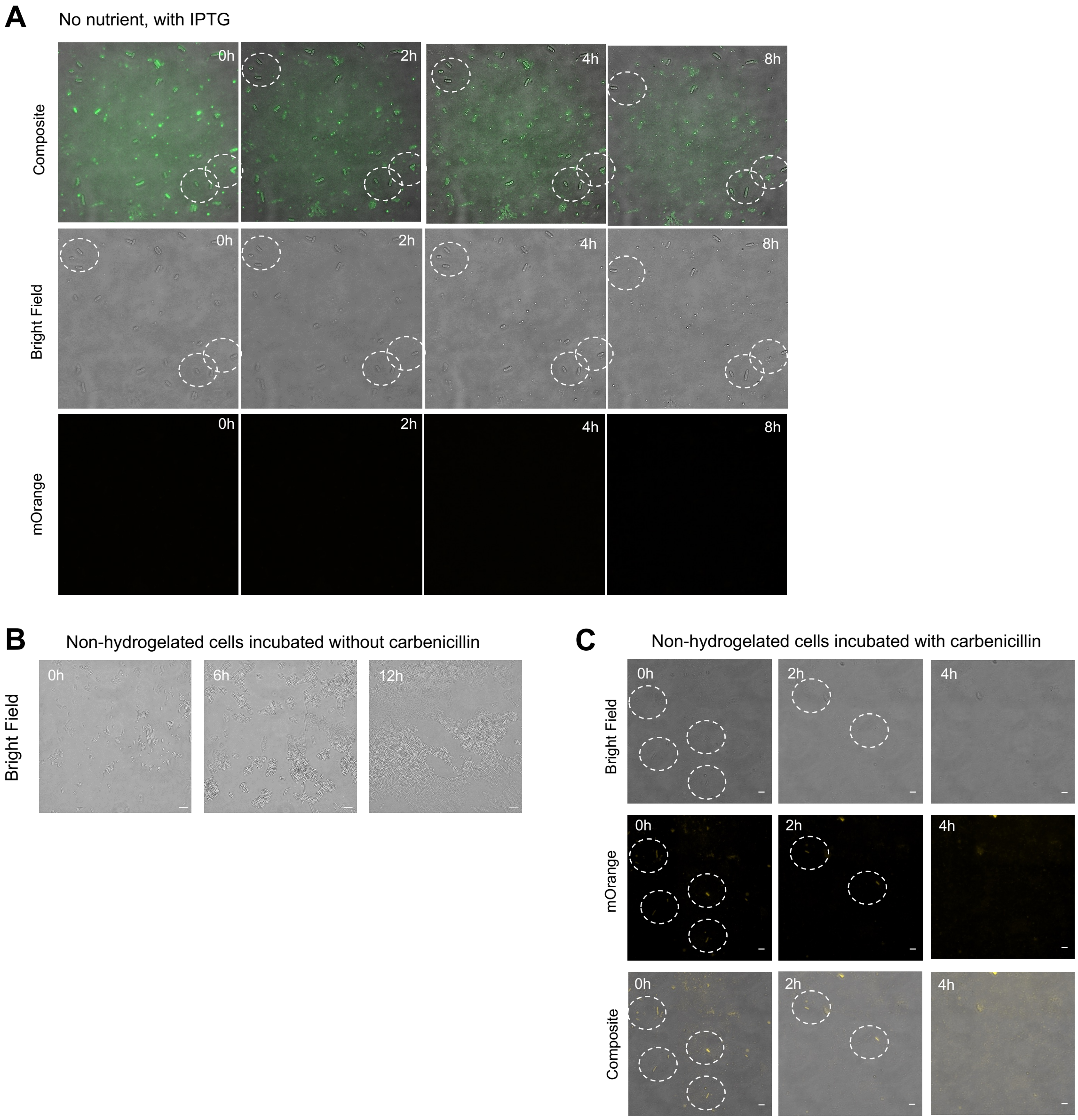

Supplementary Figure 9: Phenotypic characterization of Cyborg *E. coli* BL21 (DE3) Cells

A) Cyborg *E. coli* BL21 (DE3) pLysS, pIURKL-mOrange do not express mOrange proteins when they are incubated in media without nutrients (1XPBS 1.5%LMTA). In the presence of nutrients and under the same experimental conditions, Cyborg Cells express mOrange (Fig. 2B). See Methods Section M5. Scalebar = 5 μm. **B)** Cyborg *E. coli* BL21 (DE3) Cells divide without restriction in the absence of carbenicillin. (Scalebar = 5 μm). **C)** Cyborg *E. coli* BL21 (DE3) Cells expressing mOrange undergo cellular lysis in the presence of carbenicillin. (Scalebar = 5 μm).

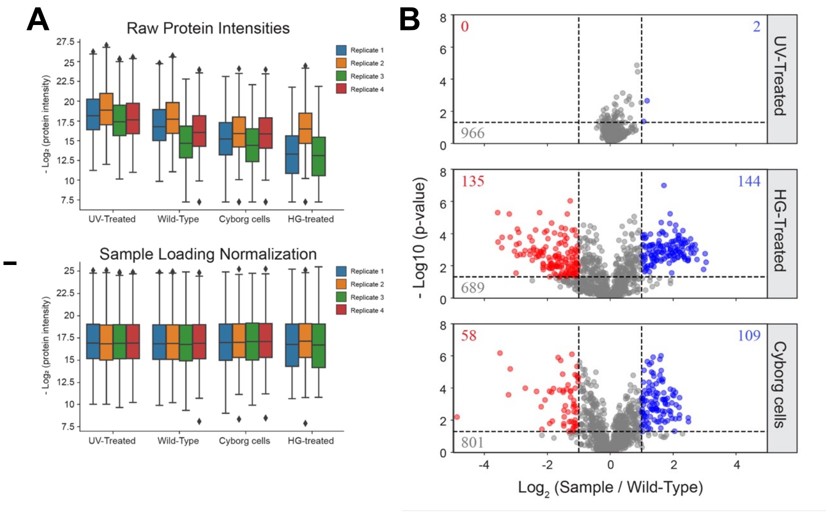

Supplementary Figure 10: Proteomic Data Analysis

A) Sample Loading Normalization. See Methods Section 12. **B)** Volcano plots showing the individual proteins being up (blue dots) and down (red dots) regulated in *E. coli* BL21 (DE3) Cells treated with only UV (UV-Treated), co-incubated with hydrogelation buffer (HG-Treated), and hydrogelated (Cyborg Cells). Individual proteins compared to the mean value in Wild Type Cells.

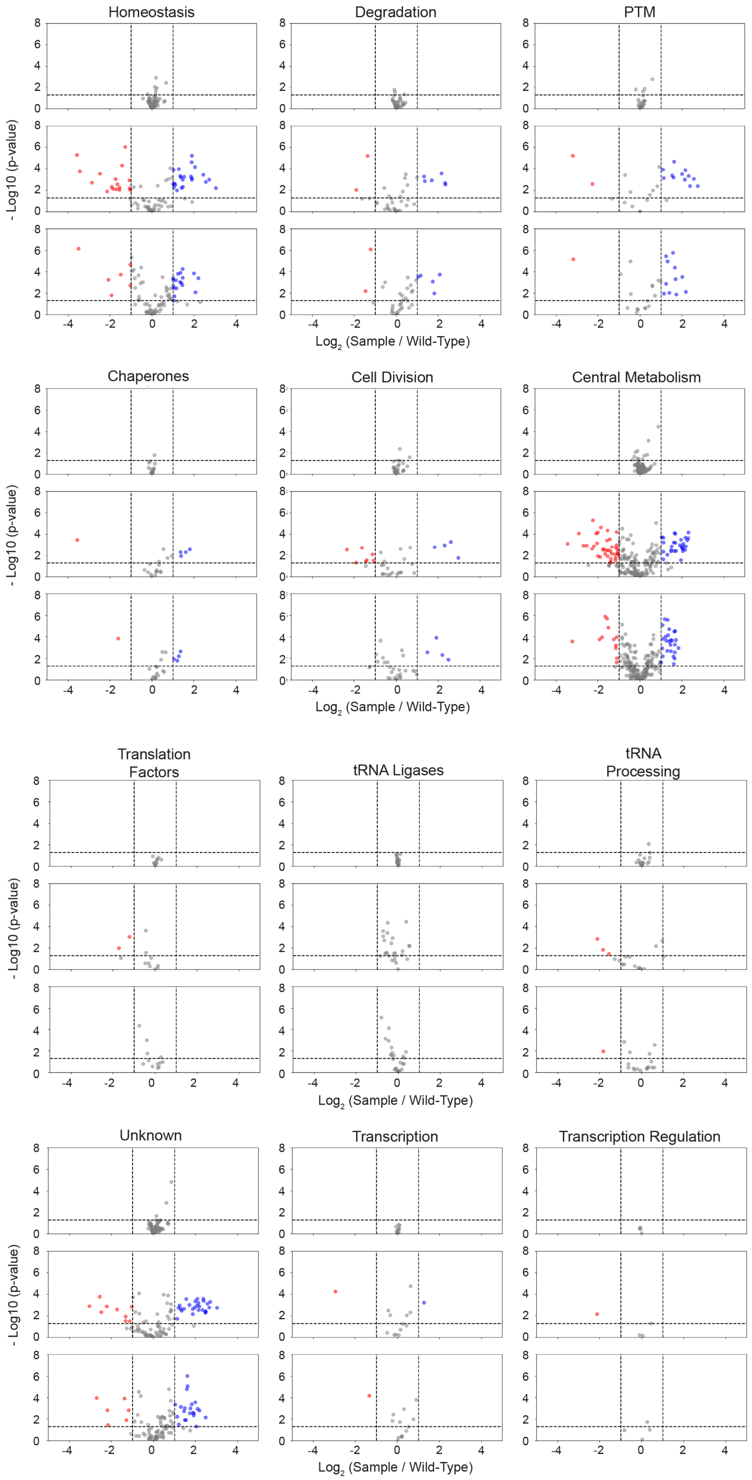
Homeostasis
Degradation
PTM
Chaperones
Cell Division
Central Metabolism
Translation Factors
tRNA Ligases
tRNA Processing
Unknown
Transcription
Transcription Regulation
- Log10 (p-value)
Log2 (Sample / Wild-Type)

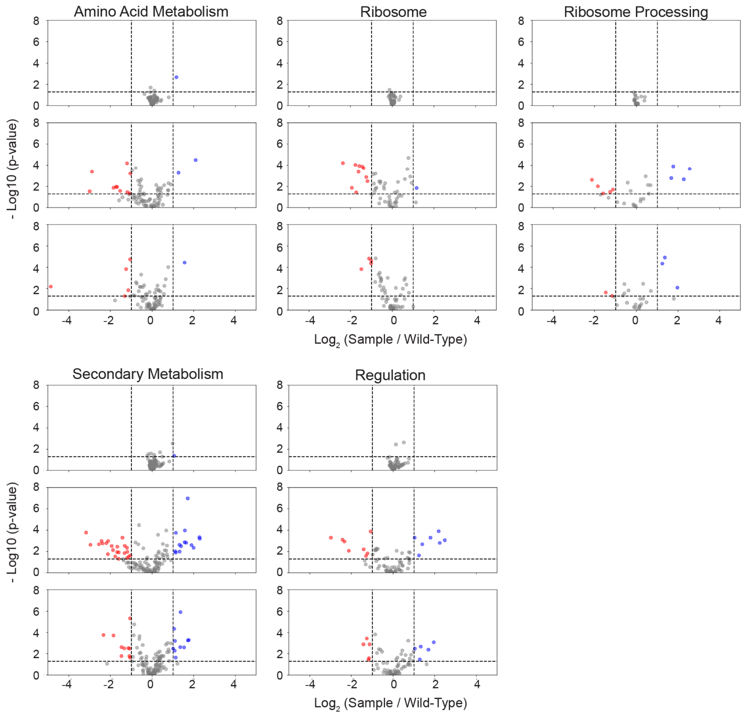

Supplementary Figure 11: Analysis of Individual Functional Protein Groups

Volcano plots showing individual proteins that are up (blue dots) and down (red dots) regulated in *E. coli* BL21 (DE3) Cells (UV-Treated, HG-Treated, and Cyborg Cells) across 17 different functional protein groups. Top panels: UV-treated. Middle panels: HG-treated. Bottom panels: Cyborg Cells.

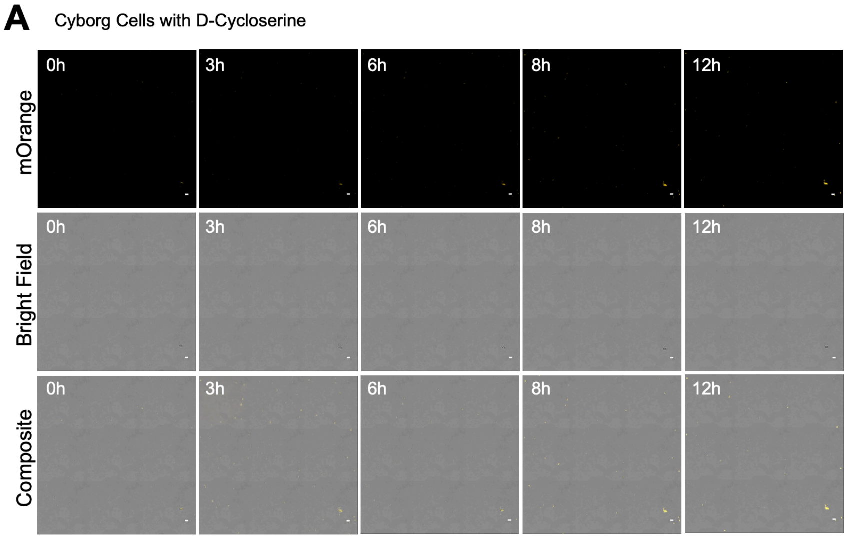

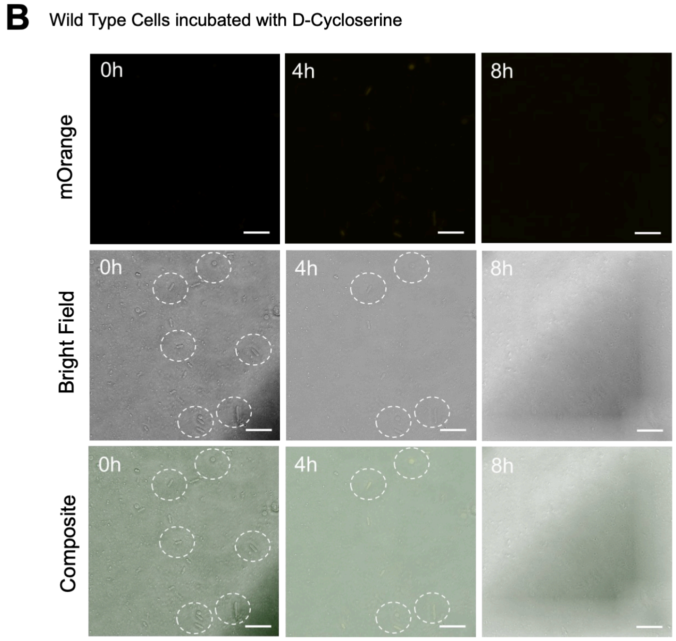

Supplementary Figure 12: Cyborg Cells remain functional with D-Cycloserine treatment
A) Cyborg Cells incubated in a media containing D-Cycloserine express mOrange in response to IPTG induction. **B)** Wild Type Cells incubated in a media containing D-Cycloserine are lysed. Scalebar = 5 μm.

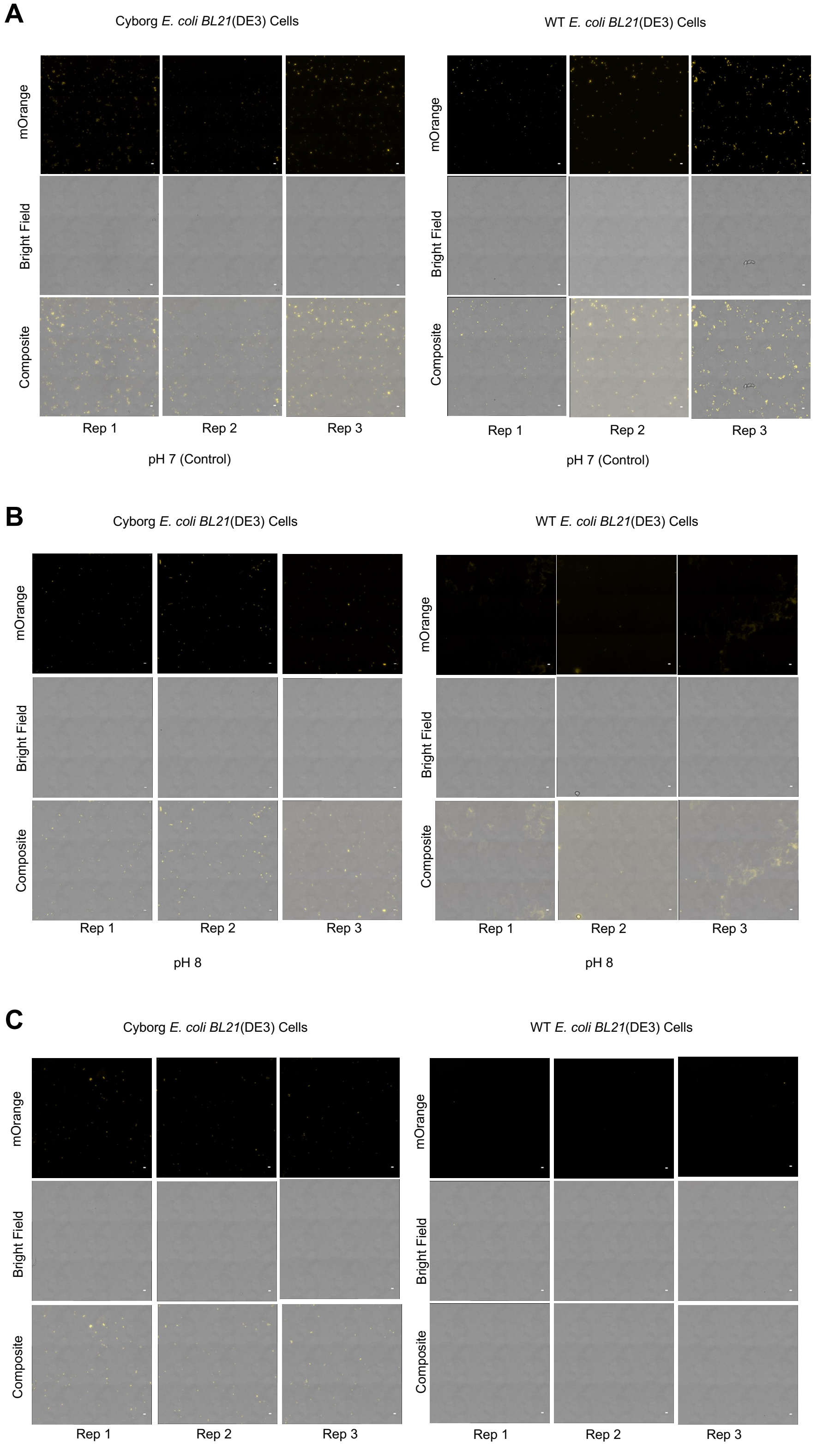

Supplementary Figure 13: Cyborg *E. coli* BL21(DE3) Cells remain functional at high pH

Fluorescence microscopy images showing replicates of Wild Type and Cyborg Cells incubated in a rich media with different pH values (7-9). **A)** Incubation of Wild Type and Cyborg Cells in media with pH 7. **B)** Incubation of Wild Type and Cyborg Cells in media with pH 8. **C)** Incubation of Wild Type and Cyborg Cells in media with pH 9. n = 3 independent experiments. Scalebar = 5 μm. All cells were induced with IPTG (1 mM) and incubated in a media at the specific pH at the same time.

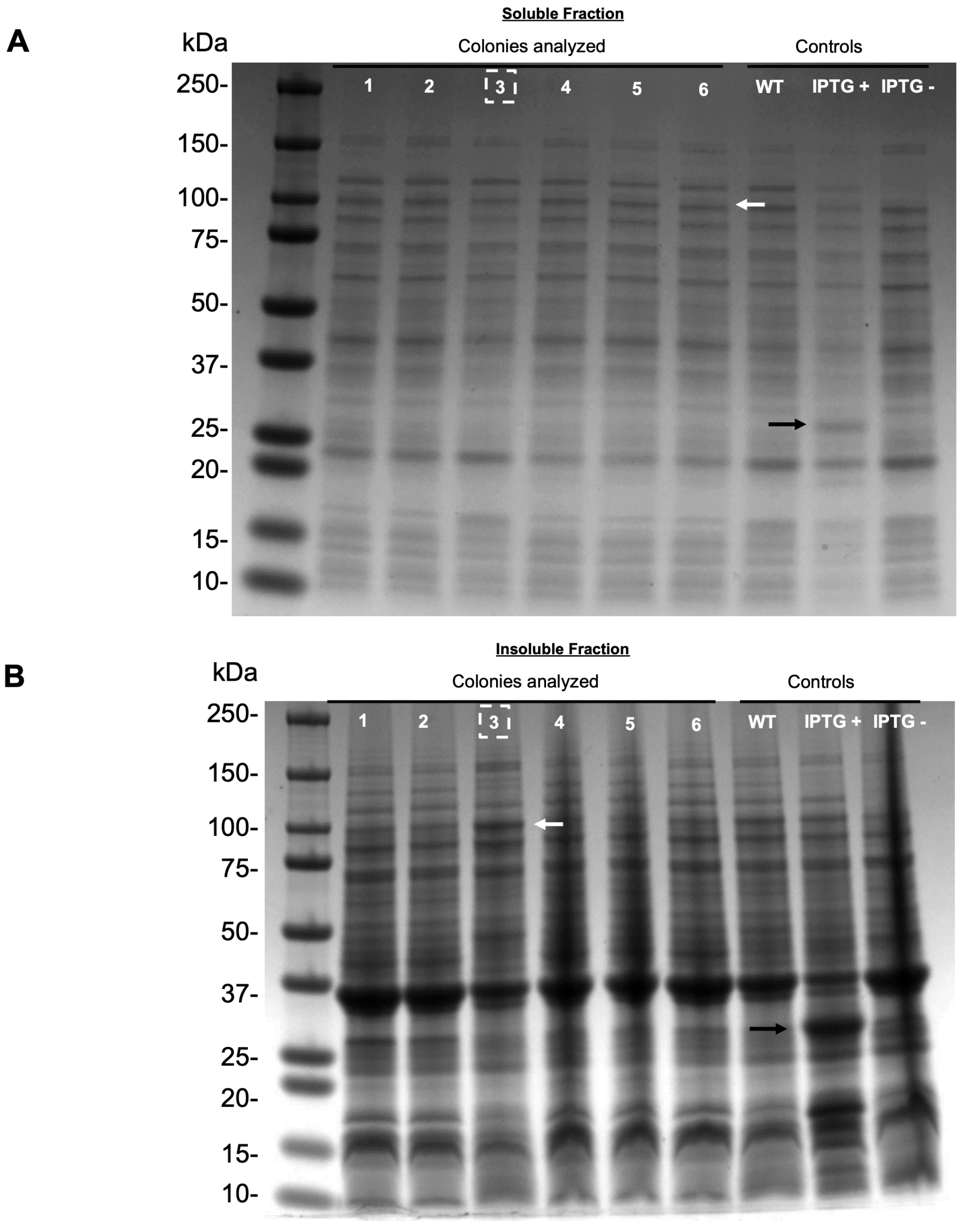

Supplementary Figure 14: SDS PAGE analysis of *E. coli* expressing Invasin

A) Analysis of the soluble fraction of 6 different clones. **B)** Analysis of the insoluble fraction of 6 different clones showing invasin expression. Arrows show the location of the expected invasin (top), and mOrange bands in both gels.

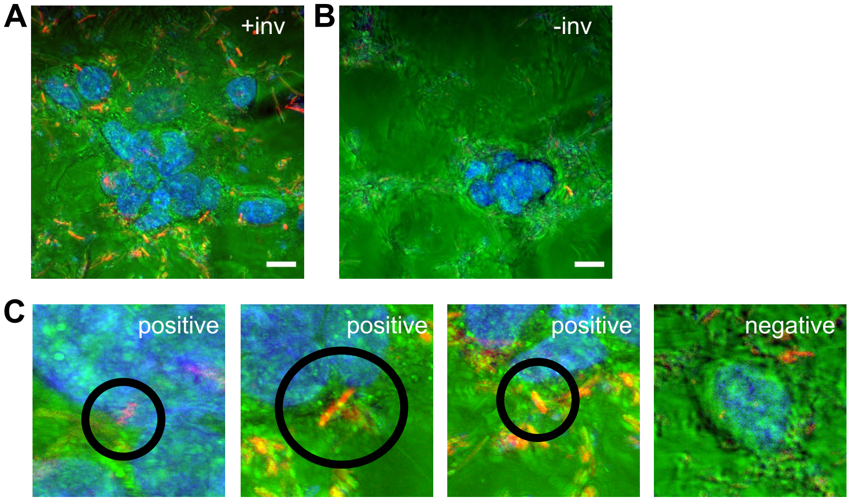

Supplementary Figure 15: Analysis of the invasion of Cyborg Cells into cancer cells

A&B) Additional fake colorized microscope images of SH-SY5Y incubated with Cyborg *E. coli* BL21(DE3) Cells expressing invasin and mOrange (**A**), and mOrange only (**B**) Scalebar = 10μm. **C)** Images used for training observers in the blind counting test. Positive=invasion. Negative=no invasion. See Methods Section M14.

Section C: Supplementary Movies

Supplementary Video 1: Real-time tracking of Wild Type BL21DE3

Supplementary Video 2: Real-time tracking of Cyborg BL21DE3

Supplementary Video 3: Real-time tracking of Wild Type MG1655

Supplementary Video 4: Real-time tracking of Cyborg MG1655
